# Altered Phenotype and Enhanced Antibody-Producing Ability of Peripheral B Cells in Mice with *Cd19*-Driven Cre Expression

**DOI:** 10.3390/cells11040700

**Published:** 2022-02-16

**Authors:** Ying Zhao, Sai Zhao, Xiao-Yuan Qin, Ting-Ting He, Miao-Miao Hu, Zheng Gong, Hong-Min Wang, Fang-Yuan Gong, Xiao-Ming Gao, Jun Wang

**Affiliations:** 1 Department of Pathophysiology, School of Biology and Basic Medical Sciences, Soochow University, Suzhou 215123, China; yzhao@suda.edu.cn; 2 Institutes of Biology and Medical Sciences, Soochow University, Suzhou 215123, China; 20194252021@stu.suda.edu.cn (S.Z.); xiaoyuan_qin@junshipharma.com (X.-Y.Q.); he_tingting0801@wuxiapptec.com (T.-T.H.); 20204250091@stu.suda.edu.cn (M.-M.H.); 20184052010@stu.suda.edu.cn (Z.G.); hmwang@suda.edu.cn (H.-M.W.); gongfangyuan@suda.edu.cn (F.-Y.G.)

**Keywords:** B cells, *Cd19^Cre/+^* mice, humoral responses, CD19 expression, inflammation

## Abstract

Given the importance of B lymphocytes in inflammation and immune defense against pathogens, mice transgenic for Cre under the control of *Cd19* promoter (*Cd19^Cre/+^* mice) have been widely used to specifically investigate the role of *loxP*-flanked genes in B cell development/function. However, impacts of expression/insertion of the Cre transgene on the phenotype and function of B cells have not been carefully studied. Here, we show that the number of marginal zone B and B1a cells was selectively reduced in *Cd19^Cre/+^* mice, while B cell development in the bone marrow and total numbers of peripheral B cells were comparable between *Cd19^Cre/+^* and wild type C57BL/6 mice. Notably, humoral responses to both T cell-dependent and independent antigens were significantly increased in *Cd19^Cre/+^* mice. We speculate that these differences are mainly attributable to reduced surface CD19 levels caused by integration of the Cre-expressing cassette that inactivates one *Cd19* allele. Moreover, our literature survey showed that expression of *Cd19^Cre/+^* alone may affect the development/progression of inflammatory and anti-infectious responses. Thus, our results have important implications for the design and interpretation of results on gene functions specifically targeted in B cells in the *Cd19^Cre/+^* mouse strain, for instance, in the context of (auto) inflammatory/infectious diseases.

## 1. Introduction

The Cre/*loxP* recombination system has been widely used to edit mammalian genomes in genetic and biomedical studies. Upon recognizing the 34-bp-long *loxP* motif inserted at defined positions of the genome, the recombinase Cre specifically and efficiently drives the recombination of DNA segments flanked by two *loxP* recognition sites (‘floxed’ locus) [1]. Hence, the regulation of Cre expression with inducible or cell/tissue-specific promoters represents an elegant and powerful approach to precisely interrogate the function of genes that have been inactivated or activated in a spatially and temporally specific fashion [2]. In particular, the Cre/*loxP*-mediated conditional-knockout system circumvents the indispensability of certain genes in embryonic development, and enables the elucidation of their functions in specific organs or cells in vivo.

Despite the widespread usage in experimental studies, the Cre/*loxP* system has a number of limitations/shortcomings [2,3]. Apart from the variable excision efficiencies of floxed loci, off-target Cre expressions/activities have been reported in several Cre lines, including CD11c-Cre and Thy1-Cre, where a wide range of cells/tissues are targeted beyond expectation [4,5,6]. Moreover, given that the mammalian genome comprises many cryptic/pseudo *loxP* sites, for instance, at an estimated frequency of 1.2 per megabase in the mouse genome, the mere expression of Cre is potentially toxic to cells and thus may result in reduced proliferation, aberrant DNA recombination, and chromosomal defects [7,8,9,10,11]. For instance, the expression of Cre driven by the promoter of *Lck* (encoding the protein tyrosine kinase p56) in thymocytes significantly reduces thymic cellularity and promotes the apoptosis of CD4^+^CD8^+^ double-positive T cells [12]. In addition to the toxicity inherent to Cre recombinase, insertion of the Cre transgene into the genome may affect the expression of endogenous genes around the integration site through direct disruption of their sequences, and/or the *trans*-effect mediated by control elements present in the transgenic vector (i.e., promoters & enhancers, etc.) [13,14]. Interestingly, most Cre knock-in mouse strains seem to tolerate these aforementioned adverse effects, possibly through developmental selection and adaptation processes, and appear to be phenotypically indistinguishable from wild-type (WT) controls as described in the literature [2,3]. Therefore, inclusion of mice or cells expressing the Cre transgene only as a critical control is frequently ignored in the field, which may lead to misinterpretation of the data on gene functions.

The type I transmembrane protein CD19 is first upregulated in the bone marrow (BM) pro-B cells, and thereafter its surface density is maintained at high levels throughout B cell development and maturation stages until the terminally differentiated plasma cells (PCs) arise [15,16]. As such, CD19 is one of the most reliable surface lineage markers for B cells, and transgenic mice with Cre expression under the control of *Cd19* promoter represent an excellent model to elucidate the roles of different genes in B cell development, differentiation, and function at steady states and/or in the context of infectious/inflammatory disorders. However, considering the disruption of one *Cd19* allele by insertion of the Cre-expressing cassette in the commonly used *Cd19^Cre/+^* hemizygotes (hereafter referred to as *Cd19^Cre/+^* mice) originally generated by Rajewsky’s group [17,18], and the involvements of CD19 in B cell signaling and function [15], we hypothesized that these *Cd19^Cre/+^* mice may differ from WT controls as a result of reduced CD19 expression and/or Cre-mediated side effects. In line with this, a few published studies showed that *Cd19*-driven expression of Cre might reduce BM pre-B cell numbers, alter follicular vs. marginal zone B (MZB) cell development, and hamper the survival of mature B cells in mice with certain genetic backgrounds [19,20,21].

Here we demonstrate that *Cd19*-driven expression of Cre alters the phenotype and function of peripheral B cells, albeit the comparable early BM B cell development, in the widely used C57BL/6 mice. The integration/expression of Cre transgene reduces the number of MZB and B1a cells, and augments antibody productions both in vivo and in vitro. Moreover, a survey of published studies using *Cd19^Cre/+^* to delete floxed-sequences in B cells indicated that results in some studies would have been less/more significant if CD19-Cre^+^, instead of CD19-Cre^−^, mice had been used as controls, validating the relevance and importance of our observations. As such, *Cd19^Cre/+^* mice or B cells are critical controls in studies using *Cd19*-driven Cre to specifically investigate gene functions in B cell biology, especially in infectious or inflammatory conditions involving innate-like MZB and B1 cells as well as antibody productions from conventional B2 cells.

## 2. Materials and Methods

### 2.1. Mice

The *Cd19^Cre/+^* mice on a C57BL/6 background, with one allele containing a *Cre* recombinase gene under the control of endogenous *Cd19* promoter/enhancer elements, were kindly provided by Prof. Biao Zheng (East China Normal University, Shanghai, China). These mice were bred with WT C57BL/6 mice to obtain *Cd19^Cre/+^* and WT (*Cd19^+/+^*) control littermates. Mice were housed under specific pathogen-free (SPF) conditions and used at 8–12 weeks of age unless otherwise indicated. All experiments were performed in accordance with procedures approved by the Animal Care and Use Committee of Soochow University.

### 2.2. Immunizations

Mice were immunized with NP-Ficoll (Biosearch Technologies, Middleton, WI, USA, 10 μg/100 μL/mouse in PBS) or NP_19_-OVA (Biosearch Technologies) adsorbed onto Imject alum (Thermo Scientific, Waltham, MA, USA, 1:1, 30 μg/100 μL/mouse) intraperitoneally.

### 2.3. Cell Isolation and Culture

BM cells were harvested by flushing the femurs of mice, and peritoneal cells were isolated by lavaging the peritoneal cavities with 10 mL PBS. Splenocytes were obtained by mechanically dissociating spleens in PBS, followed by passing then through a 70 μM nylon mesh (BD Biosciences, San Jose, CA, USA). Erythrocytes were removed by addition of ammonium chloride lysis buffer. Splenic CD19^+^ B cells were first enriched by negative selection with the MojoSort Mouse Pan B Cell Isolation Kit II (Biolegend, San Diego, CA, USA), and then were sorted out via a FACS Aria cell sorter III (BD Biosciences) with >95% purity.

Splenocytes or purified B cells were labelled with CFSE (10 μM, Thermo Scientific) before being cultured with LPS (0.1/1 μg/mL, L2630, Sigma, Saint Louis, MO, USA) or goat F(ab’)_2_ anti-mouse IgM (αIgM, 1/10 μg/mL, SouthernBiotech, Birmingham, AL, USA) plus IL-4 (20 ng/mL, Peprotech, Cranbury, NJ, USA) in 96 U-bottom plates to induce proliferations. For in vitro differentiation towards antibody-secreting cells (ASCs) and class-switch recombination (CSR) into IgG1^+^ cells, purified B cells (5 × 10^4^/well) were stimulated with LPS (10 μg/mL) ± IL-4 (25 ng/mL) in 96 U-bottom plates at 37 °C in a humidified incubator. The complete culture medium was RPMI-1640 (Hyclone, Logan, UT, USA) supplemented with 10% heat-inactivated fetal bovine serum (FBS, Gibico, Grand Island, NY, USA), 100 U/mL penicillin (Beyotime, Nantong, China), 100 μg/mL streptomycin (Beyotime), 50 μM β-mercaptoethanol (Sigma), and 1 mM sodium pyruvate and 10 mM HEPES (Hyclone).

### 2.4. ELISA & ELISPOT Analysis

Sera or culture supernatants were collected at indicated time-points and stored at −20 °C until the analysis for antibody titers by ELISA. In brief, ELISA plates (Nunc, Waltham, MA, USA) were coated with goat anti-mouse Ig (SouthernBiotech, 1010-01, 1:1000) or NP_25_-BSA (Biosearch Technologies, 5 μg/mL) to capture all murine Igs or NP-specific antibodies, respectively. After washing with PBS containing 0.05% Tween-20, wells were blocked with PBS, containing 3% BSA, before incubation with properly diluted sera or culture supernatants. Total or NP-specific IgM, IgG1, IgG2b, IgG2c, IgG3, and IgA levels were detected by using HRP-coupled goat anti-mouse subtype-specific secondary antibodies (SouthernBiotech).

For ELISPOT assays, PVDF membranes (MSIPS4510, Millipore, Billerica, MA, USA) were coated with NP_25_-BSA (1.5 μg/100 μL/well) in PBS overnight at 4 °C, and then blocked with culture medium (RPMI-1640 + 10% FBS) for 2 h at 37 °C. Splenocytes (5 × 10^5^/100 μL/well) were added into wells and cultured in an incubator for 6 hr. After washing the PVDF membrane with PBS containing 0.05% Tween-20 3 times, cells were lysed with distilled water for 10 min on ice, followed by washing with PBS twice. Finally, membranes were stained with HRP-coupled goat anti-mouse IgM, IgG1, IgG2c, or IgG3 (1:1000, SouthernBiotech) overnight at 4°C before coloring with the AEC Substrate Set (BD Biosciences). The membranes were dried in the dark at room temperature, and spots were counted using a dissecting microscope.

### 2.5. Flow Cytometric Analysis

Single cell suspensions were prepared, and surface molecules were stained at 4 °C for 30 min with optimal dilutions of each antibody. The amounts of antibodies with minimal background staining on negative samples/cells but bright signals on positive samples/cells were chosen. The following antibodies were used: anti-mouse B220 (RA3-6B2), CD45 (30-F11), CD19 (6D5), CD23 (B3B4), CD5 (53-7.3), CD38 (90), CD184 (L276F12), CD86 (PO3), and IgG1 (RMG1-1) (all from Biolegend); anti-mouse CD21/35 (eBio4E3 or eBio8D9), CD43 (eBioR2/60), CD93 (AA4.1), IgM (II/41), and IgD (11-26c) (all from eBioscience, San Diego, CA, USA); and anti-mouse CD138 (281-2) & CD95 (Jo2) (both from BD Biosciences). Sometimes 7-AAD (Biolegend) and NP-Ficoll-FITC (NP-FITC, Biosearch Technologies) were additionally used to visualize NP-specific B cells. After staining, cells were washed twice with PBS, suspended in 300 μL PBS, and fixed volumes of cells were processed with the Attune^®^ NxT Acoustic Focusing Cytometer (Thermo Scientific). Data were analyzed by FlowJo software (BD Biosciences).

### 2.6. Literature Survey

We analyzed 336 publications shown to cite the original article generating/characterizing the *Cd19^Cre/+^* mice in the PubMed website [18]. Seventy-one hits were discarded, as they were reviews/articles/book chapters that either did not include experiments with *Cd19^Cre/+^* mice or used *Cd19^Cre/Cre^* homozygotes. Moreover, 9 publications using mice harboring one copy of *Cd19^Cre/+^* transgene for lineage tracing, imaging, or inducible depletion of B cells were excluded as well. The remaining 256 articles (Supplementary File S1), in which *Cd19^Cre/+^* was used to delete loxp-flanked sequences, were included in our analysis.

### 2.7. Statistical Analysis

The Mann-Whitney test was used to compare differences among groups by using GraphPad Prism 8.0 software (GraphPad, San Diego, CA, USA) and values at *p* < 0.05 were considered significant. χ^2^ tests were employed to compare the results in the literatures using CD19-Cre^−^ or CD19-Cre^+^ mice as controls.

## 3. Results

### 3.1. Comparable B Cell Development in the BM of Cd19^Cre/+^ Mice

Although *Cd19^Cre/+^* mice, originally generated by Rajewsky’s group, were phenotypically normal and widely used to specifically delete loxp-flanked sequence in B cells in the last two decades, the Cre expression cassette was inserted into the second coding exon, where it inactivated one allele of *Cd19* and thus reduced the latter’s surface expression levels [17,18]. Given that CD19 acts as a B-cell receptor (BCR) co-receptor, and may regulate B cell development [22,23,24,25], we performed a detailed comparison on the B cell development and phenotype in WT (*Cd19^+/+^*) vs. *Cd19^Cre/+^* mice.

**Figure 1:**
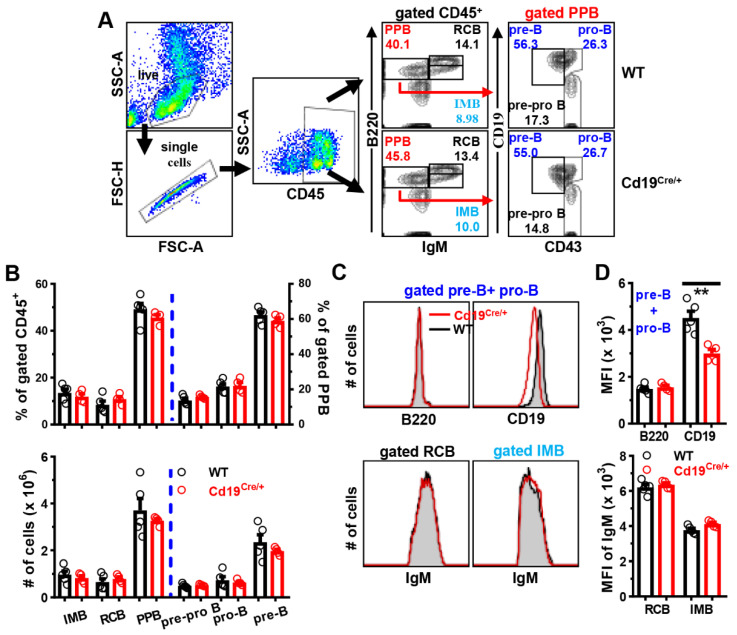
**Comparable B cell development in the BM between WT and *Cd19^Cre^^/+^*mice.** BM mononuclear cells were isolated from the femur of mice, and stained with antibodies against CD45/B220/IgM/CD19 and CD43, followed by analyses on FACS. (**A**) Representative FACS plots showing the gating strategies for pre/pro (PPB, B220^low^IgM^−^), re-circulating (RCB, B220^high^IgM^+^), and immature (IMB, B220^low^IgM^+^) B cells within gated CD45^+^ BM cells, or pre-B (CD19^+^CD43^−^), pro-B (CD19^+^CD43^+^) and pre-pro B (CD19^−^CD43^+^) cells in gated BM PPB cells between WT and Cd19^Cre/+^ mice. (**B**) Bar graphs showing the percentages (*upper panel*) or absolute numbers (*lower panel*) of indicated B cell subsets. (**C**) Representative overlayed histograms showing surface expression levels of B220 (*upper left*)/CD19 (*upper right*) on gated CD19^+^ pre-B plus pro-B cells, or IgM on gated RCB (*lower left*)/IMB (*lower right*) cells in WT (black line) and *Cd19^Cre/+^* (red line) mice. (**D**) Bar graphs showing the mean fluorescence intensity (MFI) of B220/CD19 on gated CD19^+^ pre-B plus pro-B cells (*upper panel*) or IgM on gated RCB/IMB cells in the two groups of mice (female, 8–10 weeks of age; *n* = 5/4 for WT/*Cd19^Cre/+^* group, respectively). Each symbol represents one single mouse, and results are expressed as mean ± SEM (**B**,**D**). ** *p* < 0.01.

The percentages or absolute numbers of immature (IMB, B220^low^IgM^+^), re-circulating (RCB, B220^high^IgM^+^), pre/pro (PPB, B220^low^IgM^−^) B cells did not differ between the two groups of mice. Moreover, comparable amounts of pre-pro B (B220^low^IgM^−^CD19^−^CD43^+^), pro-B (B220^low^IgM^−^CD19^+^CD43^+^) and pre-B (B220^low^IgM^−^CD19^+^CD43^−^) cells were observed between WT and Cd19^Cre/+^ mice (Figure 1A,B). As expected, insertion of the Cre cassette reduced surface CD19 levels on BM CD19^+^ B cells approximately by half, while no effect on expressions of B220 or IgM was noted (Figure 1C,D).

Therefore, we concluded that the single *Cd19^Cre/+^* knock in allele has no effect on early B cell development in the BM of mice, at least on the C57BL/6 background analyzed here.

### 3.2. Mildly Disturbed Phenotypes of B Cells in the Periphery of Cd19^Cre/+^ Mice

In the periphery, no significant differences in the absolute cell numbers were noted in the blood, spleens, mesenteric lymph nodes, Peyer’s patches and peritoneal cavities of WT vs. *Cd19^Cre/+^* mice. Within the B cell compartment in spleens, a trend of decreased IgM^high^IgD^low^ B cells, containing transitional T1 cells, B1 and MZB cells, was observed in *Cd19^Cre/+^* mice (Supplementary Figure S1A,B). Moreover, given that the frequencies of T1 (AA4.1^+^IgM^high^CD23^low^), T2 (AA4.1^+^IgM^high^CD23^high^), and T3 (AA4.1^+^IgM^low^CD23^high^) B cells, discriminated by IgM vs. CD23 expressions [26,27], did not differ significantly between these two groups of mice (Supplementary Figure S1C,D), these data suggested that Cd19^Cre/+^ mice may harbor less B1 and/or MZB cells.

**Figure 2:**
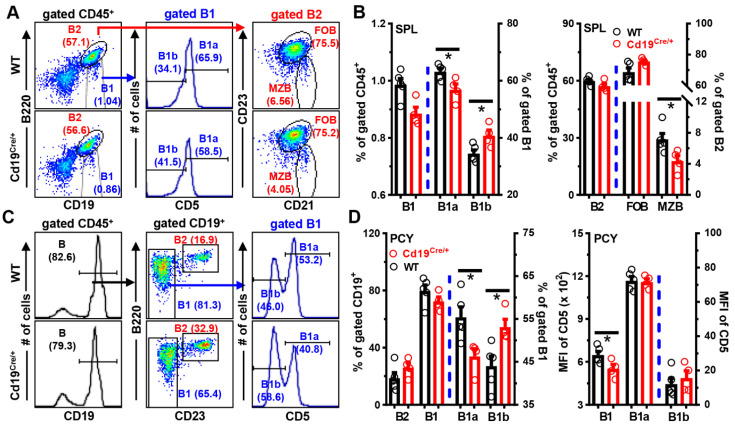
**Mildly disturbed phenotypes of B cells in the periphery of *Cd19^Cre^^/+^*mice.** Cells from the spleens (SPL) or peritoneal cavities (PCY) of mice were stained with antibodies against CD45/B220/CD19/CD5/CD21 and CD23, followed by analyses on FACS. (**A**,**C**) Representative FACS plots/histograms showing the gating strategies and percentages of B2 (CD19^+^B220^high^ in SPL or CD19^+^B220^high^CD23^+^ in PCY), B1 (CD19^+^B220^low^ in SPL or CD19^+^B220^low^CD23^−^ in PCY), B1a/b (CD5^+^/CD5^−^, respectively), follicular (FOB, CD19^+^B220^high^CD23^high^CD21^low^), and marginzal zone (MZB, CD19^+^B220^high^CD23^low^CD21^high^) B cells within gated CD45^+^ cells in the SPL (**A**) or PCY (**C**) of WT and Cd19^Cre/+^ mice. (**B**) Bar graphs showing the percentages of B1/B2 within gated CD45^+^ cells, B1a/B1b within gated B1, or FOB/MZB within gated B2 cells in the SPL. (**D**) Bar graphs showing the percentages of indicated B cell subsets (*left*), or the MFI of CD5 on gated B1, B1a, or B1b cells (*right*) in the PCY of the two groups of mice. Each symbol represents one single mouse, and results are expressed as mean ± SEM (**B**,**D**). * *p* < 0.05.

Indeed, the number of B1 cells tended to decrease in both the spleens and peritoneal cavities of *Cd19^Cre/+^* mice (Figure 2 and Figure S1E,F). In gated B1 cells, the percentage of CD5^−^ B1b cells increased at the expense of CD5^+^ B1a cells in the spleen (Figure 2A,B), peritoneal cavity (Figure 2C,D), and peripheral blood (data not shown) of *Cd19^Cre/+^* mice. Moreover, surface levels of CD5 on peritoneal total B1 cells, but not gated B1a or B1b cells, were significantly reduced in *Cd19^Cre/+^* mice (Figure 2D), indicating that the attenuated CD5 expressions on peritoneal *Cd19^Cre/+^* B1 cells mainly resulted from an altered distribution of B1a vs. B1b subsets rather than a general decrease of surface CD5. Notably, only the numbers of B1a, but not B1b, cells were reduced in the spleen and peritoneal cavity of *Cd19^Cre/+^* mice (Supplementary Figure S1E,F). In addition, numbers of MZB cells were significantly reduced in *Cd19^Cre/+^* mice (Figure 2A,B and Figure S1E), possibly due to the reduced surface CD19 intensities [23,25,28]. Interestingly, the frequencies of B1 and B1a cells returned to normal, while MZB cells remained decreased in older (16 weeks) *Cd19^Cre/+^* mice (Supplementary Figure S1G).

Together, these data show that, despite the normal BM B cell development, *Cd19^Cre/+^* mice exhibit a mildly decreased generation/maintenance of B1a cells early in life (<3 months old), and a reduced number of MZB cells at least until 16 weeks of age (Figure 1 and Figure 2).

### 3.3. Increased Antibody Levels in Cd19^Cre/+^ Mice upon Immunization

**Figure 3:**
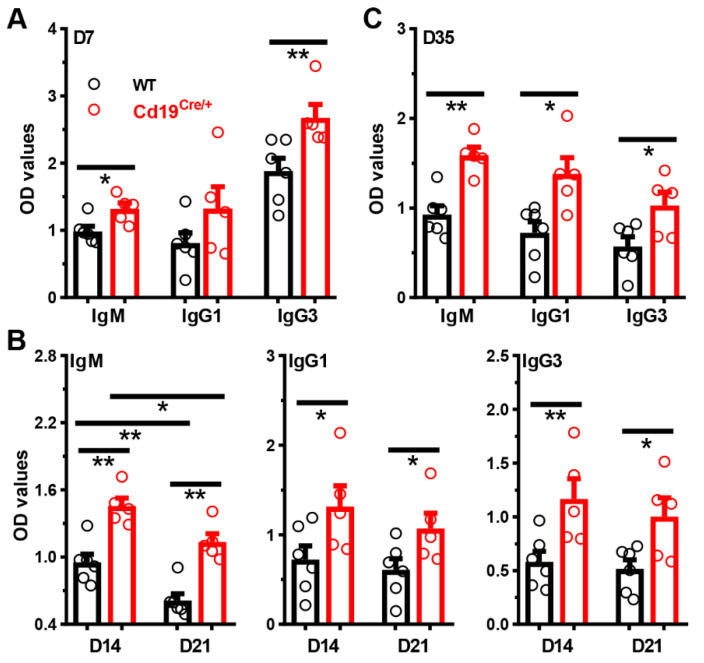
**Significantly increased antigen-specific antibody levels in *Cd19^Cre^^/+^*mice following immunization with NP-Ficoll.** Mice (female, ~10 weeks of age) were immunized with NP-Ficoll (10 μg/100 μL/mouse in PBS) intraperitoneally on D0. Levels of NP-specific IgM, IgG1 and IgG3 in sera of *Cd19^Cre/+^* or control WT littermates on D7 (**A**), D14/D21 (**B**), and D35 (**C**) post-immunization were determined by ELISA. Each symbol represents a single mouse of the indicated genotype, and results are expressed as mean ± SEM. * *p* < 0.05; ** *p* < 0.01.

Considering the roles of B1 and MZB cells in the production of protecting antibodies, we compared the total or antigen-specific sera antibody levels in *Cd19^Cre/+^* and control WT littermates before and after immunizations. The baseline antibody levels did not differ significantly between these two groups of mice (Supplementary Figure S2A). However, after immunization with the T cell independent type II antigen (TI-II-Ag) NP-Ficoll, *Cd19^Cre/+^* mice produced significantly increased amounts of NP-specific IgM, IgG1 and IgG3, three major antibody subtypes against soluble protein or carbohydrate antigens in mice [29], at all time-points tested (Figure 3). Notably, this phenomenon was gender-independent, as similar results were observed in both female (Figure 3) and male (Supplementary Figure S2B,D) *Cd19^Cre/+^* mice. The IgM antibodies capable of binding to coated NP_25_-BSA were low and comparable, and the NP-specific IgG was absent in the sera of naive WT and *Cd19^Cre/+^* mice (Supplementary Figure S2D).

**Figure 4:**
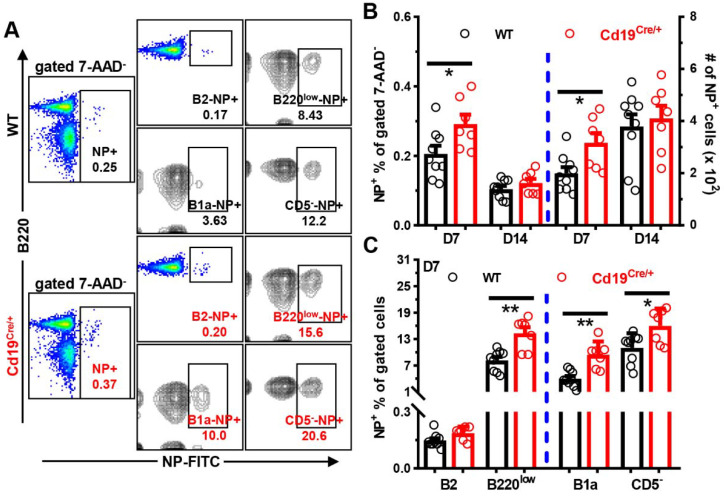
**Increased numbers of NP^+^ B cells in circulations of *Cd19^Cre^^/+^*mice immunized with NP-Ficoll.** Mice (male, ~12 weeks old) were immunized as described in the legend to Figure 3. Cells in tail blood on D7 and D14 after immunization were stained with 7-AAD and NP-FITC plus antibodies against B220/CD19/CD5. (**A**) Representative FACS plots showing the percentages of NP^+^ cells within gated live 7-AAD^−^ (NP+), CD19^+^B220^high^ B2 (B2-NP+), CD19^+^B220^low^ (B220^low^-NP+), CD19^+^B220^low^CD5^+^ B1a (B1a-NP+), and CD19^+^B220^low^CD5^−^ (CD5^−^-NP+) B cells in WT vs. *Cd19^Cre/+^* mice on D7. Gates for CD19^+^B220^low^/CD19^+^B220^low^CD5^−^ cells were equivalent to those for B1/B1b in Supplementary Figure S3A, respectively. (**B**) A bar graph showing the percentages or numbers (per 40 μL blood) of total NP^+^ cells within gated 7-AAD^−^ live cells on D7 and D14. (**C**) A bar graph showing the percentages of NP^+^ cells within gated B2, B220^low^ or B1a/B220^low^CD5^−^ cells in the two groups of mice on D7 (*n* = 9/7 for WT/Cd19^Cre/+^ group, respectively). Each symbol represents one single mouse of the indicated genotype, and results are expressed as mean ± SEM (**B**,**C**). * *p* < 0.05; ** *p* < 0.01.

**Figure 5:**
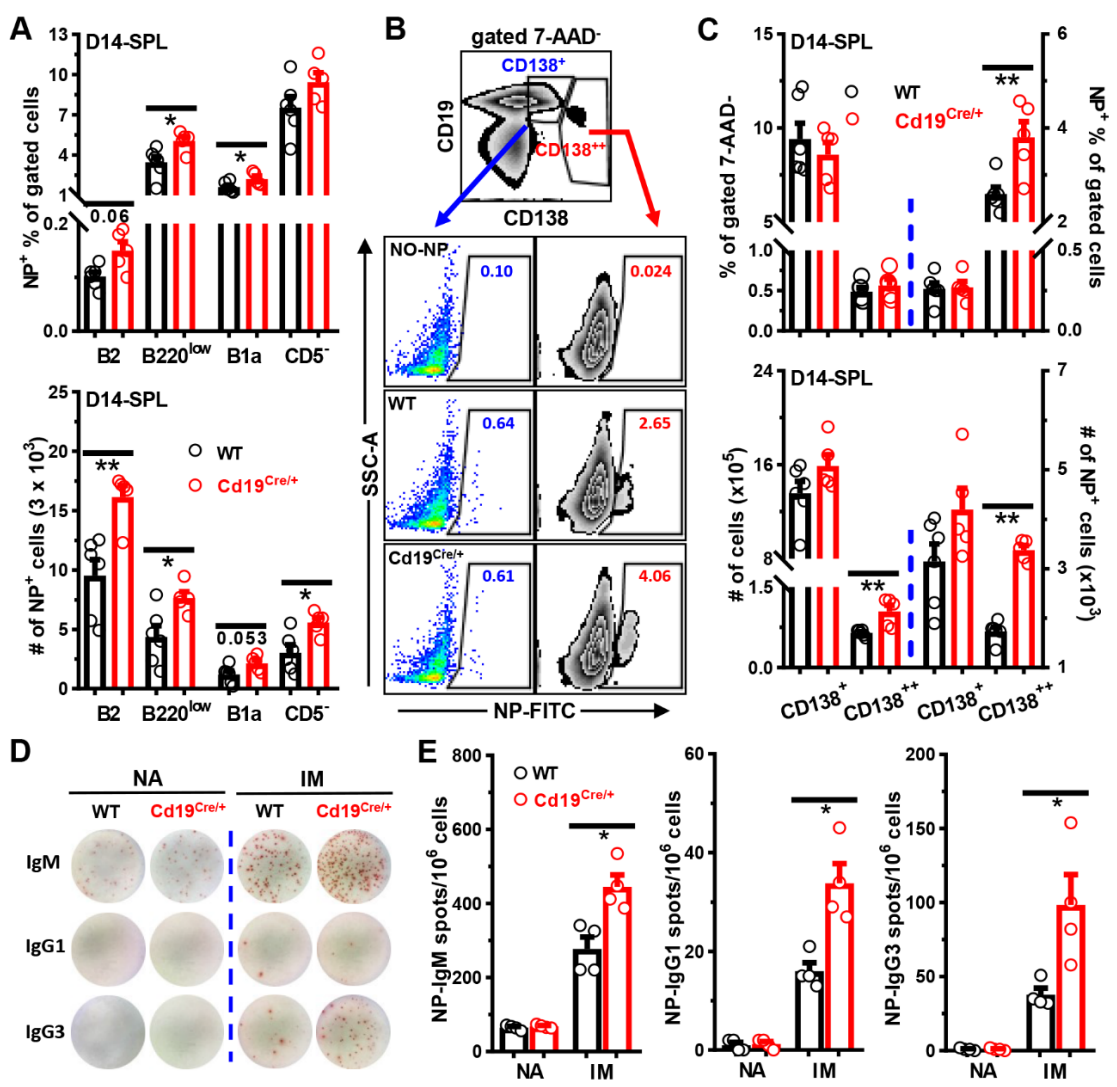
**Increased numbers of NP^+^ B cells in spleens of *Cd19^Cre^^/+^*mice immunized with NP-Ficoll.** Mice were immunized as described in the legend to Figure 3. Cells in spleens (SPL) on D14 after immunization were stained with 7-AAD, NP-FITC plus antibodies against B220/CD19/CD5 and CD138 (**A**–**C**) or cultured on NP_25_-BSA-coated PVDF membranes for ELISPOT analysis (**D**,**E**). (**A**) Bar graphs showing the percentages (*upper panel*) and numbers (*lower panel*) of NP^+^ cells within gated B2 (CD19^+^B220^high^), B220^low^ (CD19^+^B220^low^), B1a (CD19^+^B220^low^CD5^+^), and CD5^−^(CD19^+^B220^low^CD5^−^) B cells in WT vs. Cd19^Cre/+^ mice. Gates for CD19^+^B220^low^/CD19^+^B220^low^CD5^−^ cells were the same as those for B1/B1b in Figure 2A, respectively. (**B**) Representative FACS plots showing the gating strategies for CD19^+^CD138^low^ (CD138^+^), CD19^low^CD138^high^ (CD138^++^) or the percentages of NP^+^ within gated CD138^+/^CD138^++^ cells in WT vs. *Cd19^Cre/+^* mice. A sample stained with all the antibodies but without NP-FITC (NO-NP, 2nd row) served as the negative control. (**C)** Bar graphs showing the percentages (*upper panel*) and numbers (*lower panel*) of CD138^+/^CD138^++^ cells or NP^+^ cells within gated CD138^+/^CD138^++^ populations in the two groups of mice. (**D**,**E**) Representative pictures (**D**) or bar graphs (**E**) showing the numbers of NP-specific spots in spleens of naive (NA) and immunized (IM) WT vs. *Cd19^Cre/+^* mice. Each symbol represents one single male mouse of the indicated genotype, and results are expressed as mean ± SEM (**A**,**C**,**E**). * *p* < 0.05; ** *p* < 0.01.

We next stained cells in the peripheral blood, spleens, or peritoneal cavities with NP-FITC in combination with 7-AAD and antibodies to visualize NP-specific B cells. The sequential gating strategy to define NP-positivity in different B cell subsets is shown in Supplementary Figure S3A–D. The background bindings of NP-FITC to different B cell subsets were minimal and comparable between WT and *Cd19^Cre/+^* mice (Supplementary Figure S3C–E). In accordance with higher NP-specific antibody levels, *Cd19^Cre/+^* mice had more NP^+^ cells on D7 in blood post-NP-Ficoll immunization (Figure 4A,B). Within the B cell compartment, significantly increased percentages and numbers of NP-specific CD19^+^B220^low^ cells (phenotypically resembling B1 gated in Figure 2A, but containing B1, plasmablasts and pre-plasmablasts in immunized mice) were observed in blood of *Cd19^Cre/+^* mice on both D7 (Figure 4A,C) and D14 (Supplementary Figure S2C) after NP-Ficoll injection. Moreover, numbers of NP^+^ B220^high^ B2 and B220^low^ cells, as well as CD19^low^CD138^high^ ASCs, were significantly increased in the spleens of *Cd19^Cre/+^* mice two weeks after NP-Ficoll administration (Figure 5A–C). ELISPOT analyses confirmed the significantly increased amounts of NP-specific ASCs in the spleens of immunized, but not naive, *Cd19^Cre/+^* mice as well (Figure 5D,E). Even after 5 weeks, NP^+^ B220^low^ and CD19^low^CD138^high^ ASCs were still slightly increased in *Cd19^Cre/+^* mice (Supplementary Figure S4). 

**Figure 6:**
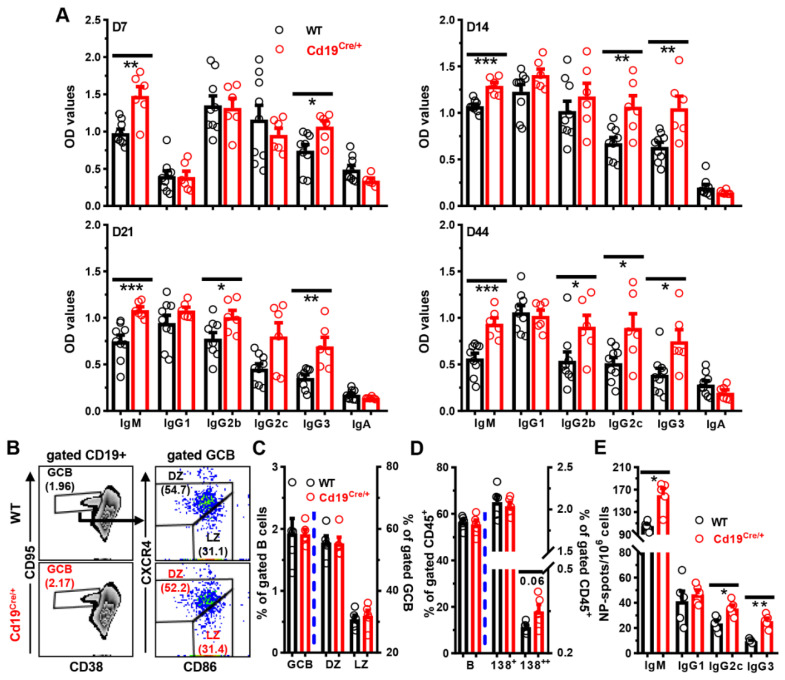
**Augmented antibody responses in *Cd19^Cre^^/+^*mice after immunization with NP-OVA.** Mice (female, 8–10 weeks of age) were immunized with alum precipitated NP_19_-OVA (1:1, 30 μg/100 μL/mouse in PBS) intraperitoneally on D0. (**A**) Bar graphs showing levels of NP-specific IgM, IgG1, IgG2b, IgG2c, IgG3, and IgA in the sera of WT or *Cd19^Cre/+^* mice on D7, D14, D21, and D44 post-immunization. (**B**) Representative FACS plots showing the percentages of germinal center B (GCB, CD19^+^CD38^low^CD95^+^), or dark zone centroblasts (DZ, CXCR4^high^CD86^low^) and light zone centrocytes (LZ, CXCR4^low^CD86^high^) within gated GCB cells. (**C**–**E**) Bar graphs showing the percentages of indicated B cell subsets (**C**,**D**) or numbers of NP-specific spots (**E**) in spleens of WT vs. *Cd19^Cre/+^* mice. Splenocytes were stained with 7-AAD plus antibodies against CD45/CD19/CD95/CD38/CXCR4/CD86 and CD138 for FACS analysis (**B**–**D**) or cultured (5 × 10^5^/100 μL/well) on NP_25_-BSA-coated PVDF membranes for ELISPOT analysis (**E**) on D14 post immunization. CD138^+^ (138^+^) and CD138^++^ (138^++^) cells were gated as described in Figure 5B. Each symbol represents a single mouse of the indicated genotype. Results are expressed as mean ± SEM. * *p* < 0.05; ** *p* < 0.01; *** *p* < 0.001.

Likewise, after immunizations with the T cell dependent antigen (TD-Ag) NP_19_-OVA, the titers of NP-specific IgM and IgG3 were persistently higher at least for 6 weeks in *Cd19^Cre/+^* mice than those in WT littermate controls, and increased levels of NP-specific IgG2b and IgG2c were detected at later time points in *Cd19^Cre/+^* mice as well (Figure 6A). Accordingly, significantly more NP-specific IgM, IgG2c, and IgG3, but not IgG1, ASCs were observed in the spleens of *Cd19^Cre/+^* mice on D14 post-immunization (Figure 6E). Frequencies of CD19^low^CD138^high^ ASCs tended to increase in *Cd19^Cre/+^* mice, while the number and phenotype of germinal center B cells were comparable between these mice (Figure 6B–D).

Thus, after immunization with TI-II-Ag or TD-Ag, *Cd19^Cre/+^* mice produce significantly increased levels of antigen-specific antibodies than their WT counterparts, which may be attributed to increased numbers of antigen-specific B/ASCs in vivo.

### 3.4. Increased Antibody-Producing Ability of B Cells from Cd19^Cre/+^ Mice upon LPS-Stimulation In Vitro

**Figure 7:**
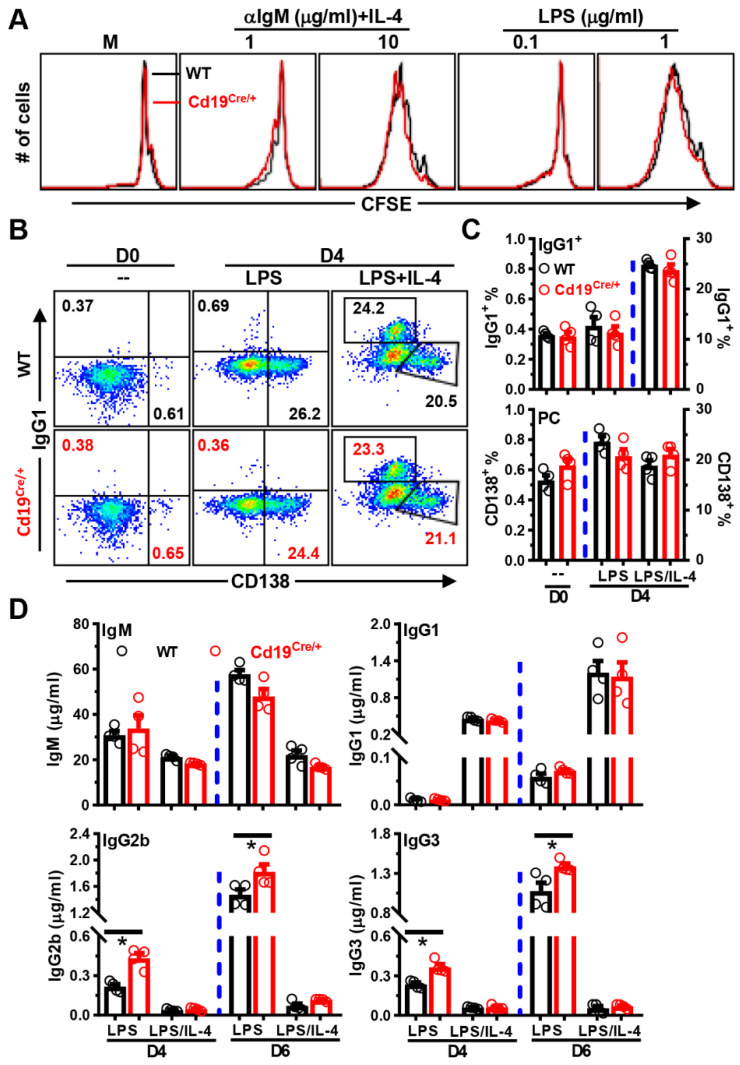
**Enhanced IgG2b and IgG3 productions of *Cd19^Cre^^/+^*B cells after stimulation with LPS in vitro.** (**A**) Representative overlayed histograms showing the CFSE profiles of gated CD19^+^ cells in different culture conditions on day 4. Splenic cells (3 × 10^5^/well) from WT or *Cd19^Cre/+^* mice were labeled with CFSE, and then stimulated with medium (M), LPS (0.1/1 μg/mL) or anti-IgM (αIgM, 1/10 μg/mL) plus IL-4 (20 ng/mL) in 96 U-bottom plates. (**B**) Representative FACS plots showing surface CD138 vs. IgG1 expressions on B cells before (D0) or after stimulation with LPS ± IL-4 for 4 days (D4). (**C**) Bar graphs showing percentages of IgG1^+^CD138^−^ (IgG1^+^) or IgG1^−^CD138^+^ plasma cells (CD138^+^, PC) on D0 or D4. (**D**) Bar graphs showing the levels of IgM/IgG1/IgG2b and IgG3 in supernatants collected on day 4 or 6. Purified CD19^+^ B cells (5 × 10^4^/well) were cultured with LPS (10 μg/mL) ± IL-4 (25 ng/mL) in 96 U-bottom plates (**B**–**D**). Each symbol represents one single mouse of the indicated genotype (female, 8–10 weeks of age, *n* = 4 for each group), and results are expressed as mean ± SEM (**C**,**D**). * *p* < 0.05.

To investigate whether the increased amounts of antigen-specific B cells in immunized *Cd19^Cre/+^* mice were attributable to increased survival/proliferation of B cells, we purified B cells from the spleens of control or *Cd19^Cre/+^* mice, and activated them with anti-IgM or LPS in the absence/presence of B cell survival factor BAFF (B cell activating factor, encoded by *Tnfsf13b*). Comparable percentages and absolute numbers of B cells were observed in cultures with purified control or *Cd19^Cre/+^* B cells, albeit the addition of BAFF significantly promoted their survivals as expected (data not shown). No differences on the expression of activation markers CD69/CD25 and costimulatory molecule CD86 were noted as well (data not shown). Moreover, after stimulating B cells with anti-IgM/LPS ± IL-4 for 4 days in vitro, the cell division profiles (Figure 7A) and absolute numbers (data not shown) of B cells did not differ significantly between WT and *Cd19^Cre/+^* mice.

We next stimulated B cells with high concentrations of LPS with/without IL-4 to induce them to differentiate into PCs and undergo CSR to IgG1^+^ cells. We observed no significant differences in the percentage of CD138^high^ PCs or class-switched IgG1^+^ B cells between WT vs. *Cd19^Cre/+^* B cells before or after culture (Figure 7B,C). Nonetheless, *Cd19^Cre/+^* B cells produced significantly higher levels of IgG2b and IgG3, but not IgM/IgG1, after stimulation with LPS on both D4 and D6 (Figure 7D), suggesting that the expression of the Cre transgene somehow promotes the CSR to IgG2b and IgG3 in LPS-stimulated B cells.

In sum, although B cells in *Cd19^Cre/+^* mice do not display an enhanced survival, proliferation, PC differentiation, or CSR to IgG1^+^ cells in our culture systems, they do produce more IgG2b and IgG3 upon LPS stimulation in vitro.

### 3.5. Survey of Published Literature Using Mice Containing Cd19^Cre/+^ Transgene Reveals That the Phenotype Is Confounded by Different Controls Used

As shown above, despite little effect on early B cell development in the BM, the single *Cd19^Cre/+^* knock in allele mildly alters the frequencies of mature B cell subsets (MZB and B1a) and elevates antibody productions both in vivo and in vitro (Figure 1, Figure 2, Figure 3, Figure 4, Figure 5, Figure 6 and Figure 7). We thus performed a survey of published articles using *Cd19^Cre/+^gene^fl/fl^* mice to study B cell-intrinsic functions of floxed genes, as we reasoned that the choice of controls (i.e., CD19-Cre^−^ vs. CD19-Cre^+^) may have biased the results/conclusions obtained.

We analyzed 256 articles where the *Cd19^Cre/+^* transgene was used to delete loxp-flanked sequence in B cells (Supplementary File S1), among which 136 studies contained the necessary information allowing for comparisons between the control (CD19-Cre^−^ or CD19-Cre^+^) and *Cd19^Cre/+^gene^fl/+^* or *Cd19^Cre/+^gene^fl/fl^* mice in at least one of the following parameters: percentages/numbers of MZB and B1a cells, or antibody levels in vivo/vitro (Supplementary Figure S5A). Similar to the phenotypes of *Cd19^Cre/+^*-transgenic mice described in this manuscript, 50% (28/56) of studies reported that CD19-Cre conditional-knock out mice/cells exhibited at least one of the following features in comparison with their CD19-Cre^−^ counterparts: reduced MZB/B1a cells or higher antibody levels in sera/culture supernatants. By contrast, only 29% (23/80) of studies using CD19-Cre^+^ controls were classified as similar. Thus, information on the functions of floxed genes deleted/overexpressed by the *Cd19*-driven Cre recombinase is significantly related to the choice of control mice used (χ^2^ test, *p* = 0.019, Supplementary Figure S5A), and it is likely that differences in some studies would have been less/more significant if CD19-Cre^+^, instead of CD19-Cre^−^, mice had been used as controls.

Moreover, within the CD19-Cre^+^-controlled group, 27 papers contained an extra CD19-Cre^−^ control (WT, *gene^fl/+^* or *gene^fl/fl^*) in some experiments, among which 12 publications reported that CD19-Cre^+^ mice were indistinguishable from their CD19-Cre^−^ counterparts in terms of the development/phenotype of B cells in the BM/periphery, and/or the function of mature B cells in vivo/vitro (Supplementary Figure S5B) [30,31,32,33,34,35,36,37,38,39,40,41]. Nevertheless, seven papers showed/indicated a significant effect of the *Cd19^Cre/+^* transgene on B cell biology and/or disease development/progression in mice [19,20,21,42,43,44,45] (Supplementary Figure S5B). Three of the seven studies explicitly reported a reduced percentage/number of MZB and/or B1a cells in *Cd19^Cre/+^* mice in comparison with CD19-Cre^−^ controls [20,21,44]. In addition, it has been shown that the *Cd19^Cre/+^* knock in allele leads to a decrease/increase of pre-B/IMB cells in the BM [19,21], an accelerated mortality in lupus-prone (NZB × NZW) F1 mice [21], a faster weight recovery post influenza infection [42], a slightly shorter life-span [43], as well as an augmented LPS-induced CSR to IgG3^+^ cells in vitro [45]. As such, the single *Cd19^Cre/+^* knock in allele, in certain contexts, has a broad and profound impact on B cell biology and beyond in mice.

## 4. Discussion

Data presented in this study show that expression of Cre under the control of *Cd19* promoter reduces the number of splenic MZB cells, alters the frequency of B1a vs. B1b cells in the periphery, and potentiates antibody productions both in vivo and in vitro after immunization/stimulation. Thus, *Cd19^Cre/+^* mice or B cells are critical controls in studies using *Cd19*-driven Cre to specifically inactivate or activate genes in B cells, as lacking these controls may lead to misinterpretation of the data and biased conclusions on gene functions in B cell biology.

Expression of the Cre recombinase alone may be toxic, possibly via recognizing cryptic/pseudo *loxP* sites in the genome, and thus significantly impacts the survival and function of mammalian cells, including T lymphocytes [7,8,9,12,46,47]. However, our results, showing the undisturbed BM B cell development, comparable total B cell numbers in the periphery, intact survival of B cells in vitro, and augmented humoral responses to both TD- and TI-II Ags in vivo, rule out a general and significant toxicity of Cre recombinase in *Cd19^Cre/+^* mice on the widely used C57BL/6 background.

Moreover, potentially altered endogenous gene expressions resulting from inserting/integrating of the Cre transgene represent another confounding factor in Cre-mediated gene editing systems. The *Cd19^Cre/+^* mice used in our study were originally generated by Rickert et al., in which the Cre-expression cassette was inserted into the second exon of *Cd19*, thereby disrupting the latter’s coding sequence [17,18]. Thus, the Cre-expressing heterozygotes (*Cd19^Cre/+^)/*homozygotes (*Cd19^Cre/Cre^*) are equivalent to the respective *Cd19* heterozygous (*Cd19^+/−^)/*homozygous (*Cd19^−/−^)* knock-out mice in terms of CD19 expression [17,18]. Although CD19 is dispensable for early B cell development in the BM, its deficiency results in a near complete loss of MZB and B1a cells in the periphery of mice [17,23,24,25,28,48,49,50]. Hence, the reduced number of MZB and B1a cells in the periphery of *Cd19^Cre/+^* mice may relate to their diminished surface CD19 expressions (Figure 1 and Figure 2). A detailed side-by-side comparison among WT, *Cd19^Cre/+^,* and *Cd19^Cre/Cre^* mice would give more insights on the effect of CD19 levels in these phenotypes.

As a type I transmembrane protein, CD19 functions as a dominant signaling component of a multimolecular complex on the surface of B cells. It acts as an essential co-receptor for BCR signal transduction by recruiting and amplifying the activation of Src-family protein tyrosine kinases Lyn and Fyn, and/or through the activation of PI3K and downstream Akt kinases [15]. Moreover, the CD19/21 complex colligates with BCR and thereby enhances B cell activation induced by antigen-bearing complement [15]. Given that MZB cells/precursors proliferate more than follicular B cells [51,52], and persist longer in the periphery without the influx of BM cells [53], it is conceivable that the CD21^high^ MZB cells rely more on CD19-propagated tonic BCR signaling for development, survival, and homeostatic proliferation in the periphery [24]. Likewise, it has been proposed that B1a, but not B1b, precursors reside almost exclusively in the fetal and neonatal liver, hence their numbers in adult mice are maintained primarily through BCR-dependent self-renewal of pre-existing cells rather than the replenishment from progenitors [54]. Surface levels of CD19 positively correlate with both the development and self-renewal of B1a cells [22,48]. As such, we speculate that the reduced MZB and B1a cells in *Cd19^Cre/+^* mice may be mainly attributed to attenuated CD19 expression/signaling. Intriguingly, the effect of *Cd19^Cre/+^* on B1/B1a cells appears to be age-dependent, as their frequencies revert to normal in mice beyond 3 months (Supplementary Figure S1G) [44], suggesting that these cells adopt compensatory mechanisms to overcome the effect of *Cd19^Cre/+^* later in vivo. Nonetheless, the potential toxic effect of Cre recombinase could not be excluded, as these in vivo long-persisting and highly proliferative B cells, especially MZB cells, might express higher levels of Cre at certain stages and/or be more sensitive to Cre-mediated DNA-damages.

In addition to development/phenotypes, B cells in *Cd19^Cre/+^* mice functionally resemble those with disrupted/reduced CD19 levels in terms of augmented responses to TI-II-Ags in vivo (Figure 3), indicating the presence of a similar cause–outcome relationship in these mice [50,55,56]. Apart from amplifying BCR signaling, CD19 may deliver a negative signal that inhibits B cell proliferation [56,57]. Therefore, it is possible that CD19-low expressing B cells in *Cd19^Cre/+^* mice are less suppressible, relative to CD19^high^ WT B cells, to the inhibitory signal afforded by CD19 upon binding to unknown ligands in vivo, thereby proliferating and differentiating more robustly following TI-II-Ag immunization (Figure 4 and Figure 5). Alternatively, it was proposed by Sato et al. that the lack of surface CD19 may upregulate the threshold of negative selection of B cells in the BM, and thereby allows a larger number of B cells with low-affinity receptors for Ficoll to mature and enter the circulation, resulting in enhanced peripheral responses in CD19^−/−^ mice [55]. However, it is unlikely that naive *Cd19^Cre/+^* mice possess more Ficoll- or NP-specific B cells, as their frequencies are comparable to those in unimmunized WT mice (Figure 5E and Figure S3E). These data also suggest that the specific increase (proliferation, selection and/or differentiation) of NP-specific B cells in immunized *Cd19^Cre/+^* mice is not due to differences in the pre-immune BCR repertoire [58].

In contrast, CD19 seems to be required for B cell responses to TD-Ags, as CD19^−/−^ mice are profoundly deficient in producing antibodies against this type of Ag [17,55,59]. Of note, the number of conventional B cells is reduced approximately by half in peripheral lymphoid tissues of CD19^−/−^ mice [49,59]. Restitution of CD19^−/−^ mice with one copy of human CD19 almost completely restores TD-Ag-induced antibody responses to WT levels without reverting the defects of B cell numbers, indicating that the expression of one allele of human *Cd19*-transgene enhances the responses of B cells against TD-Ags in vivo on a per cell basis [59]. Hence, it is conceivable that CD19, albeit being critical, fine tunes TD-Ag-induced antibody responses in vivo as well, and akin to the situations in TI-II-Ag immunized mice, the enhanced antibody responses against NP-OVA in *Cd19^Cre/+^* mice might also be attributed, at least partially, to the attenuated surface CD19 intensities on B cells (Figure 6). Moreover, it has been shown that Cd19-deficiency selectively compromises Th2-dependent isotype switching of B cells, possibly owing to the impaired Th2-B cell interaction in vivo [60,61]. The increased levels of NP-specific IgG1 in NP-Ficoll-administered, but not NP-OVA-immunized, *Cd19^Cre/+^* mice thus might relate to the greater impact of CD19 on Th2-coordinated B cell differentiations in vivo [60,61]. In addition, a few studies showed that CD19 on B cells blunts T cell responses by promoting the generation of regulatory B cells [62,63,64]; thus, reduced surface CD19 on *Cd19^Cre/+^* B cells may promote antibody productions indirectly via enhancing T cell responses in vivo. Nevertheless, the possibility that integration of the Cre-expressing cassette may render B cells hyperresponsive to both TI-II- and TD- Ags in vivo, for instance, via the *trans*-effect of control elements it contains, cannot be excluded. The increased amounts of (NP-specific) ASCs, but not germinal center B cells, in NP-OVA-immunized *Cd19^Cre/+^* mice suggest that the integration of Cre cassette and/or low levels of CD19 preferentially promote B cell differentiation after TD-Ag immunization.

*Cd19^Cre/+^* B cells appear to have selectively increased ability to produce IgG2b and IgG3 upon LPS activation in vitro, because their proliferation, PC differentiation, IL-4-induced CSR to IgG1, and secretions of IgM and IgG1 are all comparable to those of WT B cells (Figure 7). In line with our observations, data from a recent publication showed that LPS-induced CSR to IgG3, but not LPS/IL-4-triggered CSR to IgG1, is enhanced in *Cd19^Cre/+^* B cells [45]. Although the underlying mechanisms remain unknown, we hypothesize that expression of the Cre recombinase might somehow promote CSR to IgG2b and IgG3 in B cells under certain conditions, for instance, via inducing DNA recombination in adjacent regions. Thereafter, these antigen-specific IgG3 antibodies might promote other IgG productions by complexing and facilitating the transportation of TD-Ag to splenic follicles [65].

Notably, our findings described here indicate that *Cd19^Cre/+^* mice are critical controls in studies where Cre driven by the *Cd19* promoter is used to ablate sequences in B cells, as the lack of this control may affect the interpretation of data and thus bias the conclusions. Indeed, the literature survey we performed to compare results from different studies using CD19-Cre^−^ or CD19-Cre^+^ mice as controls indicated that conclusions were confounded by the types of control mice used (Supplementary Figure S5A). In addition to parameters measured in this article, results in CD19-Cre^+^-uncontrolled studies investigating the roles of genes in B cells in other contexts, such as infections, autoimmune diseases or inflammatory disorders, should also be considered with caution. For instance, studies have shown that *Cd19^Cre/+^* mice have a faster weight recovery post influenza infection, exhibit an exaggerated lupus development/mortality, or live slightly shorter when compared to their CD19-Cre^−^ counterparts [21,42,43].

Intriguingly, most investigators reported no differences between *Cd19^Cre/+^* and Cd19^+/+^ mice [30,31,32,33,34,35,36,37,38,39,40,41], whereas seven others did [19,20,21,42,43,44,45], among which three documented a similar effect of the Cre-transgene on MZB and B1a cells as described in this article (Supplementary Figure S5B) [20,21,44]. In contrast to the observed normal BM B cell development in *Cd19^Cre/+^* mice in most studies, two groups reported a reduced pre-B cell compartment in lupus-prone (NZB x NZW) F1 mice or Eμ-Myc C57BL/6 mice (overexpressing the c-Myc transgene under control of the immunoglobulin heavy chain gene enhancer Eμ) [19,21]. Likewise, although we observed no differences in the survival of B cells between *Cd19^Cre/+^* and WT C57BL/6 mice (data not shown), a slightly increased apoptosis was noted in CD19-Cre^+^ lupus-prone (NZB x NZW) F1 mice [21]. Therefore, impacts of the *Cd19*-driven Cre-transgene on B cell biology appear to be partially dependent on the genetic background and/or the inflammatory environment of mice. Molecular mechanisms underlying the effects of *Cd19^Cre/+^*-transgene on B cell biology merit further investigations.

## 5. Conclusions

We have demonstrated a mildly perturbed phenotype and significantly augmented antibody-secreting ability of peripheral B cells in *Cd19^Cre/+^* mice on the widely used C57BL/6 background. Thus, *Cd19^Cre/+^* mice are critical controls in studies using *Cd19^Cre/+^* to investigate gene functions in B cells, especially in the context of inflammation and infection with the involvement of innate-like MZB/B1 cells as well as antibody productions from conventional B2 cells.

## Data Availability

Not applicable.

## Supplementary Materials

The following supporting information can be downloaded at: https://www.mdpi.com/article/10.3390/cells11040700/s1, Supplementary File S1: Lists of articles surveyed. Supplementary File S2 containing: (1) Supplementary Figure S1. Disturbed phenotypes of peripheral B cells in *Cd19^Cre/+^* mice; (2) Supplementary Figure S2. Normal baseline sera antibody levels but enhanced humoral immune responses after immunization in *Cd19^Cre/+^* mice; (3) Supplementary Figure S3. Comparable background staining of NP-Ficoll in unimmunized WT vs. *Cd19^Cre/+^* mice; (4) Supplementary Figure S4. Increased numbers of NP^+^ B cells in spleens of *Cd19^Cre/+^* mice immunized with NP-Ficoll; and Supplementary Figure S5. Survey of studies.

## Abbreviations

WT, wild-type; BM, bone marrow; PCs, plasma cells; MZB, marginal B cells; ASCs, antibody-secreting cells; CSR, class-switch recombination; NP-FITC, NP-Ficoll-FITC; IMB, immature B cells; RCB, re-circulating B cells; PPB, pre/pro B cells; TI-II-Ag, T cell independent type II antigen; TD-Ag, T cell dependent antigen.

## References

[1] Orban P.C., Chui D., Marth J.D. (1992). Tissue- and site-specific DNA recombination in transgenic mice. Proc. Natl. Acad. Sci. USA.

[2] McLellan M.A., Rosenthal N.A., Pinto A.R. (2017). Cre-loxP-Mediated Recombination: General Principles and Experimental Considerations. Curr. Protoc. Mouse Biol..

[3] Schmidt-Supprian M., Rajewsky K. (2007). Vagaries of conditional gene targeting. Nat. Immunol..

[4] Abram C.L., Roberge G.L., Hu Y., Lowell C.A. (2014). Comparative analysis of the efficiency and specificity of myeloid-Cre deleting strains using ROSA-EYFP reporter mice. J. Immunol. Methods.

[5] Caton M.L., Smith-Raska M.R., Reizis B. (2007). Notch-RBP-J signaling controls the homeostasis of CD8- dendritic cells in the spleen. J. Exp. Med..

[6] Heffner C.S., Herbert Pratt C., Babiuk R.P., Sharma Y., Rockwood S.F., Donahue L.R., Eppig J.T., Murray S.A. (2012). Supporting conditional mouse mutagenesis with a comprehensive cre characterization resource. Nat. Commun..

[7] Thyagarajan B., Guimaraes M.J., Groth A.C., Calos M.P. (2000). Mammalian genomes contain active recombinase recognition sites. Gene.

[8] Semprini S., Troup T.J., Kotelevtseva N., King K., Davis J.R., Mullins L.J., Chapman K.E., Dunbar D.R., Mullins J.J. (2007). Cryptic loxP sites in mammalian genomes: Genome-wide distribution and relevance for the efficiency of BAC/PAC recombineering techniques. Nucleic Acids Res..

[9] Loonstra A., Vooijs M., Beverloo H.B., Allak B.A., van Drunen E., Kanaar R., Berns A., Jonkers J. (2001). Growth inhibition and DNA damage induced by Cre recombinase in mammalian cells. Proc. Natl. Acad. Sci. USA.

[10] Bersell K., Choudhury S., Mollova M., Polizzotti B.D., Ganapathy B., Walsh S., Wadugu B., Arab S., Kuhn B. (2013). Moderate and high amounts of tamoxifen in alphaMHC-MerCreMer mice induce a DNA damage response, leading to heart failure and death. Dis. Model. Mech..

[11] Pugach E.K., Richmond P.A., Azofeifa J.G., Dowell R.D., Leinwand L.A. (2015). Prolonged Cre expression driven by the alpha-myosin heavy chain promoter can be cardiotoxic. J. Mol. Cell. Cardiol..

[12] Carow B., Gao Y., Coquet J., Reilly M., Rottenberg M.E. (2016). lck-Driven Cre Expression Alters T Cell Development in the Thymus and the Frequencies and Functions of Peripheral T Cell Subsets. J. Immunol..

[13] Lee J.Y., Ristow M., Lin X., White M.F., Magnuson M.A., Hennighausen L. (2006). RIP-Cre revisited, evidence for impairments of pancreatic beta-cell function. J. Biol. Chem..

[14] Teitelman G., Kedees M. (2015). Mouse insulin cells expressing an inducible RIPCre transgene are functionally impaired. J. Biol. Chem..

[15] Wang K., Wei G., Liu D. (2012). CD19: A biomarker for B cell development, lymphoma diagnosis and therapy. Exp. Hematol. Oncol..

[16] Zhu C., Chen G., Zhao Y., Gao X.M., Wang J. (2018). Regulation of the Development and Function of B Cells by ZBTB Transcription Factors. Front. Immunol..

[17] Rickert R.C., Rajewsky K., Roes J. (1995). Impairment of T-cell-dependent B-cell responses and B-1 cell development in CD19-deficient mice. Nature.

[18] Rickert R.C., Roes J., Rajewsky K. (1997). B lymphocyte-specific, Cre-mediated mutagenesis in mice. Nucleic Acids Res..

[19] Grabow S., Kelly G.L., Delbridge A.R., Kelly P.N., Bouillet P., Adams J.M., Strasser A. (2016). Critical B-lymphoid cell intrinsic role of endogenous MCL-1 in c-MYC-induced lymphomagenesis. Cell Death Dis..

[20] Hart G.T., Wang X., Hogquist K.A., Jameson S.C. (2011). Kruppel-like factor 2 (KLF2) regulates B-cell reactivity, subset differentiation, and trafficking molecule expression. Proc. Natl. Acad. Sci. USA.

[21] Tabor D.E., Gould K.A. (2017). Estrogen receptor alpha promotes lupus in (NZBxNZW)F1 mice in a B cell intrinsic manner. Clin. Immunol..

[22] Krop I., de Fougerolles A.R., Hardy R.R., Allison M., Schlissel M.S., Fearon D.T. (1996). Self-renewal of B-1 lymphocytes is dependent on CD19. Eur. J. Immunol..

[23] Martin F., Kearney J.F. (2000). Positive selection from newly formed to marginal zone B cells depends on the rate of clonal production, CD19, and btk. Immunity.

[24] Otero D.C., Anzelon A.N., Rickert R.C. (2003). CD19 function in early and late B cell development: I. Maintenance of follicular and marginal zone B cells requires CD19-dependent survival signals. J. Immunol..

[25] You Y., Zhao H., Wang Y., Carter R.H. (2009). Cutting edge: Primary and secondary effects of CD19 deficiency on cells of the marginal zone. J. Immunol..

[26] Chung J.B., Silverman M., Monroe J.G. (2003). Transitional B cells: Step by step towards immune competence. Trends Immunol..

[27] Sakurai N., Maeda M., Lee S.U., Ishikawa Y., Li M., Williams J.C., Wang L., Su L., Suzuki M., Saito T.I. (2011). The LRF transcription factor regulates mature B cell development and the germinal center response in mice. J. Clin. Investig..

[28] Wang Y., Brooks S.R., Li X., Anzelon A.N., Rickert R.C., Carter R.H. (2002). The physiologic role of CD19 cytoplasmic tyrosines. Immunity.

[29] Ferrante A., Beard L.J., Feldman R.G. (1990). IgG subclass distribution of antibodies to bacterial and viral antigens. Pediatr. Infect. Dis. J..

[30] Arenzana T.L., Smith-Raska M.R., Reizis B. (2009). Transcription factor Zfx controls BCR-induced proliferation and survival of B lymphocytes. Blood.

[31] Homig-Holzel C., Hojer C., Rastelli J., Casola S., Strobl L.J., Muller W., Quintanilla-Martinez L., Gewies A., Ruland J., Rajewsky K. (2008). Constitutive CD40 signaling in B cells selectively activates the noncanonical NF-kappaB pathway and promotes lymphomagenesis. J. Exp. Med..

[32] Kobayashi T., Kim T.S., Jacob A., Walsh M.C., Kadono Y., Fuentes-Panana E., Yoshioka T., Yoshimura A., Yamamoto M., Kaisho T. (2009). TRAF6 is required for generation of the B-1a B cell compartment as well as T cell-dependent and -independent humoral immune responses. PLoS ONE.

[33] Park S.Y., Wolfram P., Canty K., Harley B., Nombela-Arrieta C., Pivarnik G., Manis J., Beggs H.E., Silberstein L.E. (2013). Focal adhesion kinase regulates the localization and retention of pro-B cells in bone marrow microenvironments. J. Immunol..

[34] Ramon S., Bancos S., Thatcher T.H., Murant T.I., Moshkani S., Sahler J.M., Bottaro A., Sime P.J., Phipps R.P. (2012). Peroxisome proliferator-activated receptor gamma B cell-specific-deficient mice have an impaired antibody response. J. Immunol..

[35] Veillette A., Zhang S., Shi X., Dong Z., Davidson D., Zhong M.C. (2008). SAP expression in T cells, not in B cells, is required for humoral immunity. Proc. Natl. Acad. Sci. USA.

[36] Cawley K.M., Bustamante-Gomez N.C., Guha A.G., MacLeod R.S., Xiong J., Gubrij I., Liu Y., Mulkey R., Palmieri M., Thostenson J.D. (2020). Local Production of Osteoprotegerin by Osteoblasts Suppresses Bone Resorption. Cell Rep..

[37] Guo M., Price M.J., Patterson D.G., Barwick B.G., Haines R.R., Kania A.K., Bradley J.E., Randall T.D., Boss J.M., Scharer C.D. (2018). EZH2 Represses the B Cell Transcriptional Program and Regulates Antibody-Secreting Cell Metabolism and Antibody Production. J. Immunol..

[38] Murga M., Lecona E., Kamileri I., Diaz M., Lugli N., Sotiriou S.K., Anton M.E., Mendez J., Halazonetis T.D., Fernandez-Capetillo O. (2016). POLD3 Is Haploinsufficient for DNA Replication in Mice. Mol. Cell.

[39] Xiao S., Bod L., Pochet N., Kota S.B., Hu D., Madi A., Kilpatrick J., Shi J., Ho A., Zhang H. (2020). Checkpoint Receptor TIGIT Expressed on Tim-1(+) B Cells Regulates Tissue Inflammation. Cell Rep..

[40] Xu W., Fukuyama T., Ney P.A., Wang D., Rehg J., Boyd K., van Deursen J.M., Brindle P.K. (2006). Global transcriptional coactivators CREB-binding protein and p300 are highly essential collectively but not individually in peripheral B cells. Blood.

[41] Yang Y., Li X., Ma Z., Wang C., Yang Q., Byrne-Steele M., Hong R., Min Q., Zhou G., Cheng Y. (2021). CTLA-4 expression by B-1a B cells is essential for immune tolerance. Nat. Commun..

[42] Johnson J.L., Rosenthal R.L., Knox J.J., Myles A., Naradikian M.S., Madej J., Kostiv M., Rosenfeld A.M., Meng W., Christensen S.R. (2020). The Transcription Factor T-bet Resolves Memory B Cell Subsets with Distinct Tissue Distributions and Antibody Specificities in Mice and Humans. Immunity.

[43] Kasama Y., Sekiguchi S., Saito M., Tanaka K., Satoh M., Kuwahara K., Sakaguchi N., Takeya M., Hiasa Y., Kohara M. (2010). Persistent expression of the full genome of hepatitis C virus in B cells induces spontaneous development of B-cell lymphomas in vivo. Blood.

[44] Kobayashi M., Lin Y., Mishra A., Shelly C., Gao R., Reeh C.W., Wang P.Z., Xi R., Liu Y., Wenzel P. (2020). Bmi1 Maintains the Self-Renewal Property of Innate-like B Lymphocytes. J. Immunol..

[45] Yang D., Sun Y., Chen J., Zhang Y., Fan S., Huang M., Xie X., Cai Y., Shang Y., Gui T. (2020). REV7 is required for processing AID initiated DNA lesions in activated B cells. Nat. Commun..

[46] Feyerabend T.B., Weiser A., Tietz A., Stassen M., Harris N., Kopf M., Radermacher P., Moller P., Benoist C., Mathis D. (2011). Cre-mediated cell ablation contests mast cell contribution in models of antibody- and T cell-mediated autoimmunity. Immunity.

[47] Ohnmacht C., Schwartz C., Panzer M., Schiedewitz I., Naumann R., Voehringer D. (2010). Basophils orchestrate chronic allergic dermatitis and protective immunity against helminths. Immunity.

[48] Sato S., Ono N., Steeber D.A., Pisetsky D.S., Tedder T.F. (1996). CD19 regulates B lymphocyte signaling thresholds critical for the development of B-1 lineage cells and autoimmunity. J. Immunol..

[49] Engel P., Zhou L.J., Ord D.C., Sato S., Koller B., Tedder T.F. (1995). Abnormal B lymphocyte development, activation, and differentiation in mice that lack or overexpress the CD19 signal transduction molecule. Immunity.

[50] Haas K.M., Poe J.C., Steeber D.A., Tedder T.F. (2005). B-1a and B-1b cells exhibit distinct developmental requirements and have unique functional roles in innate and adaptive immunity to *S. pneumoniae*. Immunity.

[51] Oliver A.M., Martin F., Gartland G.L., Carter R.H., Kearney J.F. (1997). Marginal zone B cells exhibit unique activation, proliferative and immunoglobulin secretory responses. Eur. J. Immunol..

[52] Srivastava B., Quinn W.J., Hazard K., Erikson J., Allman D. (2005). Characterization of marginal zone B cell precursors. J. Exp. Med..

[53] Hao Z., Rajewsky K. (2001). Homeostasis of peripheral B cells in the absence of B cell influx from the bone marrow. J. Exp. Med..

[54] Tung J.W., Mrazek M.D., Yang Y., Herzenberg L.A. (2006). Phenotypically distinct B cell development pathways map to the three B cell lineages in the mouse. Proc. Natl. Acad. Sci. USA.

[55] Sato S., Steeber D.A., Tedder T.F. (1995). The CD19 signal transduction molecule is a response regulator of B-lymphocyte differentiation. Proc. Natl. Acad. Sci. USA.

[56] Tsitsikov E.N., Gutierrez-Ramos J.C., Geha R.S. (1997). Impaired CD19 expression and signaling, enhanced antibody response to type II T independent antigen and reduction of B-1 cells in CD81-deficient mice. Proc. Natl. Acad. Sci. USA.

[57] Rigley K.P., Callard R.E. (1991). Inhibition of B cell proliferation with anti-CD19 monoclonal antibodies: Anti-CD19 antibodies do not interfere with early signaling events triggered by anti-IgM or interleukin 4. Eur. J. Immunol..

[58] Pape K.A., Maul R.W., Dileepan T., Paustian A.S., Gearhart P.J., Jenkins M.K. (2018). Naive B Cells with High-Avidity Germline-Encoded Antigen Receptors Produce Persistent IgM(+) and Transient IgG(+) Memory B Cells. Immunity.

[59] Sato S., Steeber D.A., Jansen P.J., Tedder T.F. (1997). CD19 expression levels regulate B lymphocyte development: Human CD19 restores normal function in mice lacking endogenous CD19. J. Immunol..

[60] Gardby E., Chen X.J., Lycke N.Y. (2001). Impaired CD40-signalling in CD19-deficient mice selectively affects Th2-dependent isotype switching. Scand. J. Immunol..

[61] Gardby E., Lycke N.Y. (2000). CD19-deficient mice exhibit poor responsiveness to oral immunization despite evidence of unaltered total IgA levels, germinal centers and IgA-isotype switching in Peyer’s patches. Eur J. Immunol..

[62] Matsushita T., Fujimoto M., Hasegawa M., Komura K., Takehara K., Tedder T.F., Sato S. (2006). Inhibitory role of CD19 in the progression of experimental autoimmune encephalomyelitis by regulating cytokine response. Am. J. Pathol..

[63] Watanabe R., Fujimoto M., Ishiura N., Kuwano Y., Nakashima H., Yazawa N., Okochi H., Sato S., Tedder T.F., Tamaki K. (2007). CD19 expression in B cells is important for suppression of contact hypersensitivity. Am. J. Pathol..

[64] Watanabe R., Ishiura N., Nakashima H., Kuwano Y., Okochi H., Tamaki K., Sato S., Tedder T.F., Fujimoto M. (2010). Regulatory B cells (B10 cells) have a suppressive role in murine lupus: CD19 and B10 cell deficiency exacerbates systemic autoimmunity. J. Immunol..

[65] Zhang L., Ding Z., Xu H., Heyman B. (2014). Marginal zone B cells transport IgG3-immune complexes to splenic follicles. J. Immunol..

