# Supplementary material for: Altered Phenotype and Enhanced Antibody-Producing Ability of Peripheral B Cells in Mice with Cd19-Driven Cre Expression
Source: Cells. 2022 Feb 16;11(4):700. doi: 10.3390/cells11040700 (PMC8870415; doi:10.3390/cells11040700)
Supplement: Supplementary file 1 [file cells-11-00700-s001.zip › Supplementary File S1.pdf]

List of articles surveyed

I. Studies that used CD19-Cre^{-} mice as controls

1. Allende ML, Tuymetova G, Lee BG, Bonifacino E, Wu YP, Proia RL. S1P1 receptor directs the release of immature B cells from bone marrow into blood. *J Exp Med* (2010) 207(5):1113-24. doi: 10.1084/jem.20092210
2. Altuwaijri S, Chuang KH, Lai KP, Lai JJ, Lin HY, Young FM, et al. Susceptibility to autoimmunity and B cell resistance to apoptosis in mice lacking androgen receptor in B cells. *Mol Endocrinol* (2009) 23(4):444-53. doi: 10.1210/me.2008-0106
3. Anderson LJ, Longnecker R. Epstein-Barr virus latent membrane protein 2A exploits Notch1 to alter B-cell identity in vivo. *Blood* (2009) 113(1):108-16. doi: 10.1182/blood-2008-06-160937
4. Archambault AS, Carrero JA, Barnett LG, McGee NG, Sim J, Wright JO, et al. Cutting edge: Conditional MHC class II expression reveals a limited role for B cell antigen presentation in primary and secondary CD4 T cell responses. *J Immunol* (2013) 191(2):545-50. doi: 10.4049/jimmunol.1201598
5. Arcipowski KM, Stunz LL, Bishop GA. TRAF6 is a critical regulator of LMP1 functions in vivo. *Int Immunol* (2014) 26(3):149-58. doi: 10.1093/intimm/dxt052
6. Bagislar S, Sabo A, Kress TR, Doni M, Nicoli P, Campaner S, et al. Smyd2 is a Myc-regulated gene critical for MLL-AF9 induced leukemogenesis. *Oncotarget* (2016) 7(41):66398-415. doi: 10.18632/oncotarget.12012
7. Barrio L, Roman-Garcia S, Diaz-Mora E, Risco A, Jimenez-Saiz R, Carrasco YR, et al. B Cell Development and T-Dependent Antibody Response Are Regulated by p38gamma and p38delta. *Front Cell Dev Biol* (2020) 8:189. doi: 10.3389/fcell.2020.00189
8. Barwick BG, Scharer CD, Martinez RJ, Price MJ, Wein AN, Haines RR, et al. B cell activation and plasma cell differentiation are inhibited by de novo DNA methylation. *Nat Commun* (2018) 9(1):1900. doi: 10.1038/s41467-018-04234-4
9. Baschant U, Frappart L, Rauchhaus U, Bruns L, Reichardt HM, Kamradt T, et al. Glucocorticoid therapy of antigen-induced arthritis depends on the dimerized glucocorticoid receptor in T cells. *Proc Natl Acad Sci U S A* (2011) 108(48):19317-22. doi: 10.1073/pnas.1105857108
10. Broderick L, Yost S, Li D, McGeough MD, Booshehri LM, Guaderrama M, et al. Mutations in topoisomerase IIbeta result in a B cell immunodeficiency. *Nat Commun* (2019) 10(1):3644. doi: 10.1038/s41467-019-11570-6
11. Bruscoli S, Biagioli M, Sorcini D, Frammartino T, Cimino M, Sportoletti P, et al. Lack of glucocorticoid-induced leucine zipper (GILZ) deregulates B-cell survival and results in B-cell lymphocytosis in mice. *Blood* (2015) 126(15):1790-801. doi: 10.1182/blood-2015-03-631580
12. Bruscoli S, Sorcini D, Flamini S, Gagliardi A, Adamo F, Ronchetti S, et al. Glucocorticoid-Induced Leucine Zipper Inhibits Interferon-Gamma Production in B Cells and Suppresses Colitis in Mice. *Front Immunol* (2018) 9:1720. doi: 10.3389/fimmu.2018.01720
13. Cantor J, Browne CD, Ruppert R, Feral CC, Fassler R, Rickert RC, et al. CD98hc facilitates B cell proliferation and adaptive humoral immunity. *Nat Immunol* (2009) 10(4):412-9. doi: 10.1038/ni.1712
14. Carotta S, Willis SN, Hasbold J, Inouye M, Pang SH, Emslie D, et al. The transcription factors IRF8 and PU.1 negatively regulate plasma cell differentiation. *J Exp Med* (2014) 211(11):2169-81. doi: 10.1084/jem.20140425
15. Chiang YJ, Difilippantonio MJ, Tessarollo L, Morse HC, Hodes RJ. Exon 1 disruption alters tissue-specific expression of mouse p53 and results in selective development of B cell lymphomas. *PLoS One* (2012) 7(11):e49305. doi: 10.1371/journal.pone.0049305
16. Choi J, Jeon S, Choi S, Park K, Seong RH. The SWI/SNF chromatin remodeling complex regulates germinal center formation by repressing Blimp-1 expression. *Proc Natl Acad Sci U S A* (2015) 112(7):E718-27. doi: 10.1073/pnas.1418592112
17. Chu SH, Chabon JR, Matovina CN, Minehart JC, Chen BR, Zhang J, et al. Loss of H3K36 Methyltransferase SETD2 Impairs V(D)J Recombination during Lymphoid Development. *iScience* (2020) 23(3):100941. doi: 10.1016/j.isci.2020.100941
18. Ci X, Kuraoka M, Wang H, Carico Z, Hopper K, Shin J, et al. TSC1 Promotes B Cell Maturation but Is Dispensable for

Germinal Center Formation. *PLoS One* (2015) 10(5):e0127527. doi: 10.1371/journal.pone.0127527
19. Cortini A, Ellinghaus U, Malik TH, Cunninghame Graham DS, Botto M, Vyse TJ. B cell OX40L supports T follicular helper cell development and contributes to SLE pathogenesis. *Ann Rheum Dis* (2017) 76(12):2095-103. doi: 10.1136/annrheumdis-2017-211499
20. Crawford G, Hayes MD, Seoane RC, Ward S, Dalessandri T, Lai C, et al. Epithelial damage and tissue gammadelta T cells promote a unique tumor-protective IgE response. *Nat Immunol* (2018) 19(8):859-70. doi: 10.1038/s41590-018-0161-8
21. Darrah EJ, Kulinski JM, Mboko WP, Xin G, Malherbe LP, Gauld SB, et al. B Cell-Specific Expression of Ataxia-Telangiectasia Mutated Protein Kinase Promotes Chronic Gammaherpesvirus Infection. *J Virol* (2017) 91(19). doi: 10.1128/JVI.01103-17
22. Deshet-Unger N, Kolomansky A, Ben-Califa N, Hiram-Bab S, Gilboa D, Liron T, et al. Erythropoietin receptor in B cells plays a role in bone remodeling in mice. *Theranostics* (2020) 10(19):8744-56. doi: 10.7150/thno.45845
23. Detanico T, Virgen-Slane R, Steen-Fuentes S, Lin WW, Rhode-Kurnow A, Chappell E, et al. Co-expression Networks Identify DHX15 RNA Helicase as a B Cell Regulatory Factor. *Front Immunol* (2019) 10:2903. doi: 10.3389/fimmu.2019.02903
24. Didonna A, Canto Puig E, Ma Q, Matsunaga A, Ho B, Caillier SJ, et al. Ataxin-1 regulates B cell function and the severity of autoimmune experimental encephalomyelitis. *Proc Natl Acad Sci U S A* (2020) 117(38):23742-50. doi: 10.1073/pnas.2003798117
25. Duncan CG, Kondilis-Mangum HD, Grimm SA, Bushel PR, Chrysovergis K, Roberts JD, et al. Base-Resolution Analysis of DNA Methylation Patterns Downstream of Dnmt3a in Mouse Naive B Cells. *G3 (Bethesda)* (2018) 8(3):805-13. doi: 10.1534/g3.117.300446
26. Duque-Afonso J, Smith KS, Cleary ML. Conditional Expression of E2A-HLF Induces B-Cell Precursor Death and Myeloproliferative-Like Disease in Knock-In Mice. *PLoS One* (2015) 10(11):e0143216. doi: 10.1371/journal.pone.0143216
27. Elgueta R, Tse D, Deharvengt SJ, Luciano MR, Carriere C, Noelle RJ, et al. Endothelial Plasmalemma Vesicle-Associated Protein Regulates the Homeostasis of Splenic Immature B Cells and B-1 B Cells. *J Immunol* (2016) 197(10):3970-81. doi: 10.4049/jimmunol.1501859
28. Fagone P, Gunter C, Sage CR, Gunn KE, Brewer JW, Jackowski S. CTP:phosphocholine cytidylyltransferase alpha is required for B-cell proliferation and class switch recombination. *J Biol Chem* (2009) 284(11):6847-54. doi: 10.1074/jbc.M807338200
29. Fallet B, Narr K, Ertuna YI, Remy M, Sommerstein R, Cornille K, et al. Interferon-driven deletion of antiviral B cells at the onset of chronic infection. *Sci Immunol* (2016) 1(4). doi: 10.1126/sciimmunol.aah6817
30. Fernandez-Messina L, Rodriguez-Galan A, de Yebenes VG, Gutierrez-Vazquez C, Tenreiro S, Seabra MC, et al. Transfer of extracellular vesicle-microRNA controls germinal center reaction and antibody production. *EMBO Rep* (2020) 21(4):e48925. doi: 10.15252/embr.201948925
31. Flumann R, Rehkamper T, Nieper P, Pfeiffer P, Holzem A, Klein S, et al. An Autochthonous Mouse Model of Myd88- and BCL2-Driven Diffuse Large B-cell Lymphoma Reveals Actionable Molecular Vulnerabilities. *Blood Cancer Discov* (2021) 2(1):70-91. doi: 10.1158/2643-3230.BCD-19-0059
32. Fornek JL, Tygrett LT, Waldschmidt TJ, Poli V, Rickert RC, Kansas GS. Critical role for Stat3 in T-dependent terminal differentiation of IgG B cells. *Blood* (2006) 107(3):1085-91. doi: 10.1182/blood-2005-07-2871
33. Freitas do Rosario AP, Lamb T, Spence P, Stephens R, Lang A, Roers A, et al. IL-27 promotes IL-10 production by effector Th1 CD4+ T cells: a critical mechanism for protection from severe immunopathology during malaria infection. *J Immunol* (2012) 188(3):1178-90. doi: 10.4049/jimmunol.1102755
34. Frommer F, Waisman A. B cells participate in thymic negative selection of murine auto-reactive CD4+ T cells. *PLoS One* (2010) 5(10):e15372. doi: 10.1371/journal.pone.0015372
35. Fujiwara Y, Piemontese M, Liu Y, Thostenson JD, Xiong J, O'Brien CA. RANKL (Receptor Activator of NFkappaB Ligand) Produced by Osteocytes Is Required for the Increase in B Cells and Bone Loss Caused by Estrogen Deficiency in Mice. *J Biol Chem* (2016) 291(48):24838-50. doi: 10.1074/jbc.M116.742452
36. Garner OB, Yamaguchi Y, Esko JD, Videm V. Small changes in lymphocyte development and activation in mice

through tissue-specific alteration of heparan sulphate. *Immunology* (2008) 125(3):420-9. doi: 10.1111/j.1365-2567.2008.02856.x
37. Gray KS, Forrest JC, Speck SH. The de novo methyltransferases DNMT3a and DNMT3b target the murine gammaherpesvirus immediate-early gene 50 promoter during establishment of latency. *J Virol* (2010) 84(10):4946-59. doi: 10.1128/JVI.00060-10
38. Graziano VR, Alfajaro MM, Schmitz CO, Filler RB, Strine MS, Wei J, et al. CD300lf Conditional Knockout Mouse Reveals Strain-Specific Cellular Tropism of Murine Norovirus. *J Virol* (2021) 95(3). doi: 10.1128/JVI.01652-20
39. Guo F, Velu CS, Grimes HL, Zheng Y. Rho GTPase Cdc42 is essential for B-lymphocyte development and activation. *Blood* (2009) 114(14):2909-16. doi: 10.1182/blood-2009-04-214676
40. Gutjahr JC, Szenes E, Tschech L, Asslaber D, Schlederer M, Roos S, et al. Microenvironment-induced CD44v6 promotes early disease progression in chronic lymphocytic leukemia. *Blood* (2018) 131(12):1337-49. doi: 10.1182/blood-2017-08-802462
41. Hastey CJ, Ochoa J, Olsen KJ, Barthold SW, Baumgarth N. MyD88- and TRIF-independent induction of type I interferon drives naive B cell accumulation but not loss of lymph node architecture in Lyme disease. *Infect Immun* (2014) 82(4):1548-58. doi: 10.1128/IAI.00969-13
42. Horton SJ, Giotopoulos G, Yun H, Vohra S, Sheppard O, Bashford-Rogers R, et al. Early loss of Crebbp confers malignant stem cell properties on lymphoid progenitors. *Nat Cell Biol* (2017) 19(9):1093-104. doi: 10.1038/ncb3597
43. Hovelmeyer N, Wunderlich FT, Massoumi R, Jakobsen CG, Song J, Worns MA, et al. Regulation of B cell homeostasis and activation by the tumor suppressor gene CYLD. *J Exp Med* (2007) 204(11):2615-27. doi: 10.1084/jem.20070318
44. Hussain RZ, Cravens PD, Miller-Little WA, Doelger R, Granados V, Herndon E, et al. alpha4-integrin deficiency in B cells does not affect disease in a T-cell-mediated EAE disease model. *Neurol Neuroimmunol Neuroinflamm* (2019) 6(4):e563. doi: 10.1212/NXI.0000000000000563
45. Hwang SH, Lee H, Yamamoto M, Jones LA, Dayalan J, Hopkins R, et al. B cell TLR7 expression drives anti-RNA autoantibody production and exacerbates disease in systemic lupus erythematosus-prone mice. *J Immunol* (2012) 189(12):5786-96. doi: 10.4049/jimmunol.1202195
46. Ji Y, Kim H, Yang L, Sha H, Roman CA, Long Q, et al. The Sel1L-Hrd1 Endoplasmic Reticulum-Associated Degradation Complex Manages a Key Checkpoint in B Cell Development. *Cell Rep* (2016) 16(10):2630-40. doi: 10.1016/j.celrep.2016.08.003
47. Jin HY, Oda H, Chen P, Yang C, Zhou X, Kang SG, et al. Differential Sensitivity of Target Genes to Translational Repression by miR-17~92. *PLoS Genet* (2017) 13(2):e1006623. doi: 10.1371/journal.pgen.1006623
48. Jin HY, Oda H, Lai M, Skalsky RL, Bethel K, Shepherd J, et al. MicroRNA-17~92 plays a causative role in lymphomagenesis by coordinating multiple oncogenic pathways. *EMBO J* (2013) 32(17):2377-91. doi: 10.1038/emboj.2013.178
49. Jondle CN, Johnson KE, Uitenbroek AA, Sylvester PA, Nguyen C, Cui W, et al. B Cell-Intrinsic Expression of Interferon Regulatory Factor 1 Supports Chronic Murine Gammaherpesvirus 68 Infection. *J Virol* (2020) 94(13). doi: 10.1128/JVI.00399-20
50. Jones MB, Oswald DM, Joshi S, Whiteheart SW, Orlando R, Cobb BA. B-cell-independent sialylation of IgG. *Proc Natl Acad Sci U S A* (2016) 113(26):7207-12. doi: 10.1073/pnas.1523968113
51. Kirkland D, Benson A, Mirpuri J, Pifer R, Hou B, DeFranco AL, et al. B cell-intrinsic MyD88 signaling prevents the lethal dissemination of commensal bacteria during colonic damage. *Immunity* (2012) 36(2):228-38. doi: 10.1016/j.immuni.2011.11.019
52. Kitaura Y, Jang IK, Wang Y, Han YC, Inazu T, Cadera EJ, et al. Control of the B cell-intrinsic tolerance programs by ubiquitin ligases Cbl and Cbl-b. *Immunity* (2007) 26(5):567-78. doi: 10.1016/j.immuni.2007.03.015
53. Kohlhas V, Hallek M, Nguyen PH. Constitutive activation of Lyn kinase enhances BCR responsiveness, but not the development of CLL in Emicro-TCL1 mice. *Blood Adv* (2020) 4(24):6106-16. doi: 10.1182/bloodadvances.2020002584
54. Kroll J, Shi X, Caprioli A, Liu HH, Waskow C, Lin KM, et al. The BTB-kelch protein KLHL6 is involved in B-lymphocyte antigen receptor signaling and germinal center formation. *Mol Cell Biol* (2005) 25(19):8531-40. doi:

10.1128/MCB.25.19.8531-8540.2005
55. Lawler C, Stevenson PG. Type I Interferon Signaling to Dendritic Cells Limits Murid Herpesvirus 4 Spread from the Olfactory Epithelium. *J Virol* (2017) 91(23). doi: 10.1128/JVI.00951-17
56. Lentucci C, Belkina AC, Cederquist CT, Chan M, Johnson HE, Prasad S, et al. Inhibition of Ubc13-mediated Ubiquitination by GPS2 Regulates Multiple Stages of B Cell Development. *J Biol Chem* (2017) 292(7):2754-72. doi: 10.1074/jbc.M116.755132
57. Leung WH, Tarasenko T, Biesova Z, Kole H, Walsh ER, Bolland S. Aberrant antibody affinity selection in SHIP-deficient B cells. *Eur J Immunol* (2013) 43(2):371-81. doi: 10.1002/eji.201242809
58. Li C, Irrazabal T, So CC, Berru M, Du L, Lam E, et al. The H2B deubiquitinase Usp22 promotes antibody class switch recombination by facilitating non-homologous end joining. *Nat Commun* (2018) 9(1):1006. doi: 10.1038/s41467-018-03455-x
59. Li M, Cole F, Patel DS, Misenko SM, Her J, Malhowski A, et al. 53BP1 ablation rescues genomic instability in mice expressing 'RING-less' BRCA1. *EMBO Rep* (2016) 17(11):1532-41. doi: 10.15252/embr.201642497
60. Luo W, Mayeux J, Gutierrez T, Russell L, Getahun A, Muller J, et al. A balance between B cell receptor and inhibitory receptor signaling controls plasma cell differentiation by maintaining optimal Ets1 levels. *J Immunol* (2014) 193(2):909-20. doi: 10.4049/jimmunol.1400666
61. Lyszkiewicz M, Kotlarz D, Zietara N, Brandes G, Diestelhorst J, Glage S, et al. LAMTOR2 (p14) Controls B Cell Differentiation by Orchestrating Endosomal BCR Trafficking. *Front Immunol* (2019) 10:497. doi: 10.3389/fimmu.2019.00497
62. Macias-Garcia A, Heizmann B, Sellars M, Marchal P, Dali H, Pasquali JL, et al. Ikaros Is a Negative Regulator of B1 Cell Development and Function. *J Biol Chem* (2016) 291(17):9073-86. doi: 10.1074/jbc.M115.704239
63. Madan R, Demircik F, Surianarayanan S, Allen JL, Divanovic S, Trompette A, et al. Nonredundant roles for B cell-derived IL-10 in immune counter-regulation. *J Immunol* (2009) 183(4):2312-20. doi: 10.4049/jimmunol.0900185
64. McGehee AM, Dougan SK, Klemm EJ, Shui G, Park B, Kim YM, et al. XBP-1-deficient plasmablasts show normal protein folding but altered glycosylation and lipid synthesis. *J Immunol* (2009) 183(6):3690-9. doi: 10.4049/jimmunol.0900953
65. Misenko SM, Patel DS, Her J, Bunting SF. DNA repair and cell cycle checkpoint defects in a mouse model of 'BRCAness' are partially rescued by 53BP1 deletion. *Cell Cycle* (2018) 17(7):881-91. doi: 10.1080/15384101.2018.1456295
66. Moreira-Teixeira L, Redford PS, Stavropoulos E, Ghilardi N, Maynard CL, Weaver CT, et al. T Cell-Derived IL-10 Impairs Host Resistance to Mycobacterium tuberculosis Infection. *J Immunol* (2017) 199(2):613-23. doi: 10.4049/jimmunol.1601340
67. Mortales CL, Lee SU, Demetriou M. N-Glycan Branching Is Required for Development of Mature B Cells. *J Immunol* (2020) 205(3):630-6. doi: 10.4049/jimmunol.2000101
68. Mosaheb MM, Reiser ML, Wetzler LM. Toll-Like Receptor Ligand-Based Vaccine Adjuvants Require Intact MyD88 Signaling in Antigen-Presenting Cells for Germinal Center Formation and Antibody Production. *Front Immunol* (2017) 8:225. doi: 10.3389/fimmu.2017.00225
69. Mouly E, Ghamlouch H, Della-Valle V, Scourzic L, Quivoron C, Roos-Weil D, et al. B-cell tumor development in Tet2-deficient mice. *Blood Adv* (2018) 2(6):703-14. doi: 10.1182/bloodadvances.2017014118
70. Ng CT, Oldstone MB. Infected CD8alpha- dendritic cells are the predominant source of IL-10 during establishment of persistent viral infection. *Proc Natl Acad Sci U S A* (2012) 109(35):14116-21. doi: 10.1073/pnas.1211910109
71. Nie Y, Waite J, Brewer F, Sunshine MJ, Littman DR, Zou YR. The role of CXCR4 in maintaining peripheral B cell compartments and humoral immunity. *J Exp Med* (2004) 200(9):1145-56. doi: 10.1084/jem.20041185
72. Oak JS, Chen J, Peralta RQ, Deane JA, Fruman DA. The p85beta regulatory subunit of phosphoinositide 3-kinase has unique and redundant functions in B cells. *Autoimmunity* (2009) 42(5):447-58. doi: 10.1080/08916930902911746
73. Oberdoerffer P, Kanellopoulou C, Heissmeyer V, Paeper C, Borowski C, Aifantis I, et al. Efficiency of RNA interference in the mouse hematopoietic system varies between cell types and developmental stages. *Mol Cell Biol* (2005) 25(10):3896-905. doi: 10.1128/MCB.25.10.3896-3905.2005
74. Park S, Lee S, Lee CG, Park GY, Hong H, Lee JS, et al. Capicua deficiency induces autoimmunity and promotes follicular helper T cell differentiation via derepression of ETV5. *Nat Commun* (2017) 8:16037. doi: 10.1038/ncomms16037
75. Petrenko O, Li J, Cimica V, Mena-Taboada P, Shin HY, D'Amico S, et al. IL-6 promotes MYC-induced B cell

lymphomagenesis independent of STAT3. *PLoS One* (2021) 16(3):e0247394. doi: 10.1371/journal.pone.0247394
76. Qi CF, Zhang R, Sun J, Li Z, Shin DM, Wang H, et al. Homeostatic defects in B cells deficient in the E3 ubiquitin ligase ARF-BP1 are restored by enhanced expression of MYC. *Leuk Res* (2013) 37(12):1680-9. doi: 10.1016/j.leukres.2013.09.009
77. Ramezani-Rad P, Chen C, Zhu Z, Rickert RC. Cyclin D3 Governs Clonal Expansion of Dark Zone Germinal Center B Cells. *Cell Rep* (2020) 33(7):108403. doi: 10.1016/j.celrep.2020.108403
78. Ramezani-Rad P, Leung CR, Apgar JR, Rickert RC. E3 Ubiquitin Ligase Fbw7 Regulates the Survival of Mature B Cells. *J Immunol* (2020) 204(6):1535-42. doi: 10.4049/jimmunol.1901156
79. Roberts KG, Janke LJ, Zhao Y, Seth A, Ma J, Finkelstein D, et al. ETV6-NTRK3 induces aggressive acute lymphoblastic leukemia highly sensitive to selective TRK inhibition. *Blood* (2018) 132(8):861-5. doi: 10.1182/blood-2018-05-849554
80. Santoni de Sio FR, Massacand J, Barde I, Offner S, Corsinotti A, Kapopoulou A, et al. KAP1 regulates gene networks controlling mouse B-lymphoid cell differentiation and function. *Blood* (2012) 119(20):4675-85. doi: 10.1182/blood-2011-12-401117
81. Savitsky D, Calame K. B-1 B lymphocytes require Blimp-1 for immunoglobulin secretion. *J Exp Med* (2006) 203(10):2305-14. doi: 10.1084/jem.20060411
82. Scharer CD, Patterson DG, Mi T, Price MJ, Hicks SL, Boss JM. Antibody-secreting cell destiny emerges during the initial stages of B-cell activation. *Nat Commun* (2020) 11(1):3989. doi: 10.1038/s41467-020-17798-x
83. Scherger AK, Al-Maarri M, Maurer HC, Schick M, Maurer S, Ollinger R, et al. Activated gp130 signaling selectively targets B cell differentiation to induce mature lymphoma and plasmacytoma. *JCI Insight* (2019) 4(15). doi: 10.1172/jci.insight.128435
84. Schwab KR, Patel SR, Dressler GR. Role of PTIP in class switch recombination and long-range chromatin interactions at the immunoglobulin heavy chain locus. *Mol Cell Biol* (2011) 31(7):1503-11. doi: 10.1128/MCB.00990-10
85. Setz CS, Hug E, Khadour A, Abdelrasoul H, Bilal M, Hobeika E, et al. PI3K-Mediated Blimp-1 Activation Controls B Cell Selection and Homeostasis. *Cell Rep* (2018) 24(2):391-405. doi: 10.1016/j.celrep.2018.06.035
86. Siegel AM, Rangaswamy US, Napier RJ, Speck SH. Blimp-1-dependent plasma cell differentiation is required for efficient maintenance of murine gammaherpesvirus latency and antiviral antibody responses. *J Virol* (2010) 84(2):674-85. doi: 10.1128/JVI.01306-09
87. Stranges PB, Watson J, Cooper CJ, Choisy-Rossi CM, Stonebraker AC, Beighton RA, et al. Elimination of antigen-presenting cells and autoreactive T cells by Fas contributes to prevention of autoimmunity. *Immunity* (2007) 26(5):629-41. doi: 10.1016/j.immuni.2007.03.016
88. Takeuchi O, Fisher J, Suh H, Harada H, Malynn BA, Korsmeyer SJ. Essential role of BAX,BAK in B cell homeostasis and prevention of autoimmune disease. *Proc Natl Acad Sci U S A* (2005) 102(32):11272-7. doi: 10.1073/pnas.0504783102
89. Teichmann LL, Kashgarian M, Weaver CT, Roers A, Muller W, Shlomchik MJ. B cell-derived IL-10 does not regulate spontaneous systemic autoimmunity in MRL.Fas(lpr) mice. *J Immunol* (2012) 188(2):678-85. doi: 10.4049/jimmunol.1102456
90. Teichmann LL, Schenten D, Medzhitov R, Kashgarian M, Shlomchik MJ. Signals via the adaptor MyD88 in B cells and DCs make distinct and synergistic contributions to immune activation and tissue damage in lupus. *Immunity* (2013) 38(3):528-40. doi: 10.1016/j.immuni.2012.11.017
91. Tilstra JS, John S, Gordon RA, Leibler C, Kashgarian M, Bastacky S, et al. B cell-intrinsic TLR9 expression is protective in murine lupus. *J Clin Invest* (2020) 130(6):3172-87. doi: 10.1172/JCI132328
92. Todd DJ, McHeyzer-Williams LJ, Kowal C, Lee AH, Volpe BT, Diamond B, et al. XBP1 governs late events in plasma cell differentiation and is not required for antigen-specific memory B cell development. *J Exp Med* (2009) 206(10):2151-9. doi: 10.1084/jem.20090738
93. Umiker BR, McDonald G, Larbi A, Medina CO, Hobeika E, Reth M, et al. Production of IgG autoantibody requires expression of activation-induced deaminase in early-developing B cells in a mouse model of SLE. *Eur J Immunol* (2014) 44(10):3093-108. doi: 10.1002/eji.201344282
94. van Blijswijk J, Schraml BU, Rogers NC, Whitney PG, Zelenay S, Acton SE, et al. Altered lymph node composition in diphtheria toxin receptor-based mouse models to ablate dendritic cells. *J Immunol* (2015) 194(1):307-15. doi:

10.4049/jimmunol.1401999
95. Watanabe M, Lu Y, Breen M, Hodes RJ. B7-CD28 co-stimulation modulates central tolerance via thymic clonal deletion and Treg generation through distinct mechanisms. *Nat Commun* (2020) 11(1):6264. doi: 10.1038/s41467-020-20070-x
96. Wilker PR, Kohyama M, Sandau MM, Albring JC, Nakagawa O, Schwarz JJ, et al. Transcription factor Mef2c is required for B cell proliferation and survival after antigen receptor stimulation. *Nat Immunol* (2008) 9(6):603-12. doi: 10.1038/ni.1609
97. Xie P, Poovassery J, Stunz LL, Smith SM, Schultz ML, Carlin LE, et al. Enhanced Toll-like receptor (TLR) responses of TNFR-associated factor 3 (TRAF3)-deficient B lymphocytes. *J Leukoc Biol* (2011) 90(6):1149-57. doi: 10.1189/jlb.0111044
98. Xie P, Stunz LL, Larison KD, Yang B, Bishop GA. Tumor necrosis factor receptor-associated factor 3 is a critical regulator of B cell homeostasis in secondary lymphoid organs. *Immunity* (2007) 27(2):253-67. doi: 10.1016/j.immuni.2007.07.012
99. Yasuda T, Saito Y, Ono C, Kawata K, Baba A, Baba Y. Generation and characterization of CD19-iCre mice as a tool for efficient and specific conditional gene targeting in B cells. *Sci Rep* (2021) 11(1):5524. doi: 10.1038/s41598-021-84786-6
100. Yu J, Zanotti S, Schilling L, Schoenherr C, Economides AN, Sanjay A, et al. Induction of the Hajdu-Cheney Syndrome Mutation in CD19 B Cells in Mice Alters B-Cell Allocation but Not Skeletal Homeostasis. *Am J Pathol* (2018) 188(6):1430-46. doi: 10.1016/j.ajpath.2018.02.010
101. Zamora-Pineda J, Kumar A, Suh JH, Zhang M, Saba JD. Dendritic cell sphingosine-1-phosphate lyase regulates thymic egress. *J Exp Med* (2016) 213(12):2773-91. doi: 10.1084/jem.20160287
102. Zane NA, Gundelach JH, Lindquist LD, Bram RJ. Essential role for CAML in follicular B cell survival and homeostasis. *J Immunol* (2012) 188(7):3009-18. doi: 10.4049/jimmunol.1101641
103. Zhang H, Rosenberg S, Coffey FJ, He YW, Manser T, Hardy RR, et al. A role for cFLIP in B cell proliferation and stress MAPK regulation. *J Immunol* (2009) 182(1):207-15. doi: 10.4049/jimmunol.182.1.207

II. Studies that used CD19-Cre^{+} mice as controls

104. Acharya M, Sokolovska A, Tam JM, Conway KL, Stefani C, Raso F, et al. alphav Integrins combine with LC3 and atg5 to regulate Toll-like receptor signalling in B cells. *Nat Commun* (2016) 7:10917. doi: 10.1038/ncomms10917
105. Adams CM, Eischen CM. Inactivation of p53 is insufficient to allow B cells and B-cell lymphomas to survive without Dicer. *Cancer Res* (2014) 74(14):3923-34. doi: 10.1158/0008-5472.CAN-13-1866
106. Alankus B, Ecker V, Vahl N, Braun M, Weichert W, Macher-Goppinger S, et al. Pathological RANK signaling in B cells drives autoimmunity and chronic lymphocytic leukemia. *J Exp Med* (2021) 218(2). doi: 10.1084/jem.20200517
107. Alari-Pahissa E, Grandclement C, Jeevan-Raj B, Leclercq G, Veillette A, Held W. Activation by SLAM Family Receptors Contributes to NK Cell Mediated "Missing-Self" Recognition. *PLoS One* (2016) 11(4):e0153236. doi: 10.1371/journal.pone.0153236
108. An L, Wang Y, Liu Y, Yang X, Liu C, Hu Z, et al. Rad9 is required for B cell proliferation and immunoglobulin class switch recombination. *J Biol Chem* (2010) 285(46):35267-73. doi: 10.1074/jbc.M110.161208
109. Arenzana TL, Smith-Raska MR, Reizis B. Transcription factor Zfx controls BCR-induced proliferation and survival of B lymphocytes. *Blood* (2009) 113(23):5857-67. doi: 10.1182/blood-2008-11-188888
110. Arrate MP, Vincent T, Odvody J, Kar R, Jones SN, Eischen CM. MicroRNA biogenesis is required for Myc-induced B-cell lymphoma development and survival. *Cancer Res* (2010) 70(14):6083-92. doi: 10.1158/0008-5472.CAN-09-4736
111. Ballon G, Chen K, Perez R, Tam W, Cesarman E. Kaposi sarcoma herpesvirus (KSHV) vFLIP oncoprotein induces B cell transdifferentiation and tumorigenesis in mice. *J Clin Invest* (2011) 121(3):1141-53. doi: 10.1172/JCI44417
112. Barnett LG, Simkins HM, Barnett BE, Korn LL, Johnson AL, Wherry EJ, et al. B cell antigen presentation in the initiation of follicular helper T cell and germinal center differentiation. *J Immunol* (2014) 192(8):3607-17. doi: 10.4049/jimmunol.1301284
113. Belver L, de Yebenes VG, Ramiro AR. MicroRNAs prevent the generation of autoreactive antibodies. *Immunity* (2010) 33(5):713-22. doi: 10.1016/j.immuni.2010.11.010

114. Bertossi A, Aichinger M, Sansonetti P, Lech M, Neff F, Pal M, et al. Loss of Roquin induces early death and immune deregulation but not autoimmunity. *J Exp Med* (2011) 208(9):1749-56. doi: 10.1084/jem.20110578
115. Bothmer A, Rommel PC, Gazumyan A, Polato F, Reczek CR, Muellenbeck MF, et al. Mechanism of DNA resection during intrachromosomal recombination and immunoglobulin class switching. *J Exp Med* (2013) 210(1):115-23. doi: 10.1084/jem.20121975
116. Broske AM, Vockentanz L, Kharazi S, Huska MR, Mancini E, Scheller M, et al. DNA methylation protects hematopoietic stem cell multipotency from myeloerythroid restriction. *Nat Genet* (2009) 41(11):1207-15. doi: 10.1038/ng.463
117. Browne CD, Del Nagro CJ, Cato MH, Dengler HS, Rickert RC. Suppression of phosphatidylinositol 3,4,5-trisphosphate production is a key determinant of B cell anergy. *Immunity* (2009) 31(5):749-60. doi: 10.1016/j.immuni.2009.08.026
118. Browne CD, Hoefer MM, Chintalapati SK, Cato MH, Wallez Y, Ostertag DV, et al. SHEP1 partners with CasL to promote marginal zone B-cell maturation. *Proc Natl Acad Sci U S A* (2010) 107(44):18944-9. doi: 10.1073/pnas.1007558107
119. Cawley KM, Bustamante-Gomez NC, Guha AG, MacLeod RS, Xiong J, Gubrij I, et al. Local Production of Osteoprotegerin by Osteoblasts Suppresses Bone Resorption. *Cell Rep* (2020) 32(10):108052. doi: 10.1016/j.celrep.2020.108052
120. Chandra A, van Maldegem F, Andrews S, Neuberger MS, Rada C. Deficiency in spliceosome-associated factor CTNNBL1 does not affect ongoing cell cycling but delays exit from quiescence and results in embryonic lethality in mice. *Cell Cycle* (2013) 12(5):732-42. doi: 10.4161/cc.23594
121. Chen J, Limon JJ, Blanc C, Peng SL, Fruman DA. Foxo1 regulates marginal zone B-cell development. *Eur J Immunol* (2010) 40(7):1890-6. doi: 10.1002/eji.200939817
122. Chen S, Miyazaki M, Chandra V, Fisch KM, Chang AN, Murre C. Id3 Orchestrates Germinal Center B Cell Development. *Mol Cell Biol* (2016) 36(20):2543-52. doi: 10.1128/MCB.00150-16
123. Courties G, Frodermann V, Honold L, Zheng Y, Herisson F, Schloss MJ, et al. Glucocorticoids Regulate Bone Marrow B Lymphopoiesis After Stroke. *Circ Res* (2019) 124(9):1372-85. doi: 10.1161/CIRCRESAHA.118.314518
124. Cumpelik A, Heja D, Hu Y, Varano G, Ordikhani F, Roberto MP, et al. Dynamic regulation of B cell complement signaling is integral to germinal center responses. *Nat Immunol* (2021) 22(6):757-68. doi: 10.1038/s41590-021-00926-0
125. Daly J, Bebenek K, Watt DL, Richter K, Jiang C, Zhao ML, et al. Altered Ig hypermutation pattern and frequency in complementary mouse models of DNA polymerase zeta activity. *J Immunol* (2012) 188(11):5528-37. doi: 10.4049/jimmunol.1102629
126. Daniel JA, Pellegrini M, Lee BS, Guo Z, Filsuf D, Belkina NV, et al. Loss of ATM kinase activity leads to embryonic lethality in mice. *J Cell Biol* (2012) 198(3):295-304. doi: 10.1083/jcb.201204035
127. De Silva NS, Anderson MM, Carette A, Silva K, Heise N, Bhagat G, et al. Transcription factors of the alternative NF-kappaB pathway are required for germinal center B-cell development. *Proc Natl Acad Sci U S A* (2016) 113(32):9063-8. doi: 10.1073/pnas.1602728113
128. De Silva NS, Silva K, Anderson MM, Bhagat G, Klein U. Impairment of Mature B Cell Maintenance upon Combined Deletion of the Alternative NF-kappaB Transcription Factors RELB and NF-kappaB2 in B Cells. *J Immunol* (2016) 196(6):2591-601. doi: 10.4049/jimmunol.1501120
129. Delgado P, Alvarez-Prado AF, Marina-Zarate E, Sernandez IV, Mur SM, de la Barrera J, et al. Interplay between UNG and AID governs intratumoral heterogeneity in mature B cell lymphoma. *PLoS Genet* (2020) 16(12):e1008960. doi: 10.1371/journal.pgen.1008960
130. Delgado-Benito V, Berruezo-Llacuna M, Altwasser R, Winkler W, Sundaravinayagam D, Balasubramanian S, et al. PDGFA-associated protein 1 protects mature B lymphocytes from stress-induced cell death and promotes antibody gene diversification. *J Exp Med* (2020) 217(10). doi: 10.1084/jem.20200137
131. Delgado-Benito V, Rosen DB, Wang Q, Gazumyan A, Pai JA, Oliveira TY, et al. The Chromatin Reader ZMYND8 Regulates Igh Enhancers to Promote Immunoglobulin Class Switch Recombination. *Mol Cell* (2018) 72(4):636-49 e8. doi: 10.1016/j.molcel.2018.08.042
132. Dengler HS, Baracho GV, Omori SA, Bruckner S, Arden KC, Castrillon DH, et al. Distinct functions for the transcription factor Foxo1 at various stages of B cell differentiation. *Nat Immunol* (2008) 9(12):1388-98. doi: 10.1038/ni.1667

133. Di Virgilio M, Callen E, Yamane A, Zhang W, Jankovic M, Gitlin AD, et al. Rif1 prevents resection of DNA breaks and promotes immunoglobulin class switching. *Science* (2013) 339(6120):711-5. doi: 10.1126/science.1230624
134. Dinkelmann M, Spehalski E, Stoneham T, Buis J, Wu Y, Sekiguchi JM, et al. Multiple functions of MRN in end-joining pathways during isotype class switching. *Nat Struct Mol Biol* (2009) 16(8):808-13. doi: 10.1038/nsmb.1639
135. Dolezal E, Infantino S, Drepper F, Borsig T, Singh A, Wossning T, et al. The BTG2-PRMT1 module limits pre-B cell expansion by regulating the CDK4-Cyclin-D3 complex. *Nat Immunol* (2017) 18(8):911-20. doi: 10.1038/ni.3774
136. Duque-Afonso J, Feng J, Scherer F, Lin CH, Wong SH, Wang Z, et al. Comparative genomics reveals multistep pathogenesis of E2A-PBX1 acute lymphoblastic leukemia. *J Clin Invest* (2015) 125(9):3667-80. doi: 10.1172/JCI81158
137. Feldhahn N, Ferretti E, Robbiani DF, Callen E, Deroubaix S, Selleri L, et al. The hSSB1 orthologue Obfc2b is essential for skeletogenesis but dispensable for the DNA damage response in vivo. *EMBO J* (2012) 31(20):4045-56. doi: 10.1038/emboj.2012.247
138. Galindo-Campos MA, Bedora-Faure M, Farres J, Lescale C, Moreno-Lama L, Martinez C, et al. Coordinated signals from the DNA repair enzymes PARP-1 and PARP-2 promotes B-cell development and function. *Cell Death Differ* (2019) 26(12):2667-81. doi: 10.1038/s41418-019-0326-5
139. Gibb DR, El Shikh M, Kang DJ, Rowe WJ, El Sayed R, Cichy J, et al. ADAM10 is essential for Notch2-dependent marginal zone B cell development and CD23 cleavage in vivo. *J Exp Med* (2010) 207(3):623-35. doi: 10.1084/jem.20091990
140. Giles AJ, Bender TP, Ravichandran KS. The adaptor protein Shc plays a key role during early B cell development. *J Immunol* (2009) 183(9):5468-76. doi: 10.4049/jimmunol.0902344
141. Goda N, Ryan HE, Khadivi B, McNulty W, Rickert RC, Johnson RS. Hypoxia-inducible factor 1alpha is essential for cell cycle arrest during hypoxia. *Mol Cell Biol* (2003) 23(1):359-69. doi: 10.1128/MCB.23.1.359-369.2003
142. Gonzalez-Martin A, Adams BD, Lai M, Shepherd J, Salvador-Bernaldez M, Salvador JM, et al. The microRNA miR-148a functions as a critical regulator of B cell tolerance and autoimmunity. *Nat Immunol* (2016) 17(4):433-40. doi: 10.1038/ni.3385
143. Grabow S, Kelly GL, Delbridge AR, Kelly PN, Bouillet P, Adams JM, et al. Critical B-lymphoid cell intrinsic role of endogenous MCL-1 in c-MYC-induced lymphomagenesis. *Cell Death Dis* (2016) 7:e2132. doi: 10.1038/cddis.2016.43
144. Guo M, Price MJ, Patterson DG, Barwick BG, Haines RR, Kania AK, et al. EZH2 Represses the B Cell Transcriptional Program and Regulates Antibody-Secreting Cell Metabolism and Antibody Production. *J Immunol* (2018) 200(3):1039-52. doi: 10.4049/jimmunol.1701470
145. Gyory I, Boller S, Nechanitzky R, Mandel E, Pott S, Liu E, et al. Transcription factor Ebf1 regulates differentiation stage-specific signaling, proliferation, and survival of B cells. *Genes Dev* (2012) 26(7):668-82. doi: 10.1101/gad.187328.112
146. Habib T, Park H, Tsang M, de Alboran IM, Nicks A, Wilson L, et al. Myc stimulates B lymphocyte differentiation and amplifies calcium signaling. *J Cell Biol* (2007) 179(4):717-31. doi: 10.1083/jcb.200704173
147. Haines RR, Barwick BG, Scharer CD, Majumder P, Randall TD, Boss JM. The Histone Demethylase LSD1 Regulates B Cell Proliferation and Plasmablast Differentiation. *J Immunol* (2018) 201(9):2799-811. doi: 10.4049/jimmunol.1800952
148. Haines RR, Scharer CD, Lobby JL, Boss JM. LSD1 Cooperates with Noncanonical NF-kappaB Signaling to Regulate Marginal Zone B Cell Development. *J Immunol* (2019) 203(7):1867-81. doi: 10.4049/jimmunol.1900654
149. Hart GT, Peery SL, Hamilton SE, Jameson SC. Cutting edge: Kruppel-like factor 2 is required for phenotypic maintenance but not development of B1 B cells. *J Immunol* (2012) 189(7):3293-7. doi: 10.4049/jimmunol.1201439
150. Hart GT, Wang X, Hogquist KA, Jameson SC. Kruppel-like factor 2 (KLF2) regulates B-cell reactivity, subset differentiation, and trafficking molecule expression. *Proc Natl Acad Sci U S A* (2011) 108(2):716-21. doi: 10.1073/pnas.1013168108
151. Heise N, De Silva NS, Silva K, Carette A, Simonetti G, Pasparakis M, et al. Germinal center B cell maintenance and differentiation are controlled by distinct NF-kappaB transcription factor subunits. *J Exp Med* (2014) 211(10):2103-18. doi: 10.1084/jem.20132613
152. Henderson RB, Grys K, Vehlow A, de Bettignies C, Zachacz A, Henley T, et al. A novel Rac-dependent checkpoint in B cell development controls entry into the splenic white pulp and cell survival. *J Exp Med* (2010) 207(4):837-53. doi: 10.1084/jem.20091489

153. Hiller B, Achleitner M, Glage S, Naumann R, Behrendt R, Roers A. Mammalian RNase H2 removes ribonucleotides from DNA to maintain genome integrity. *J Exp Med* (2012) 209(8):1419-26. doi: 10.1084/jem.20120876
154. Homig-Holzel C, Hojer C, Rastelli J, Casola S, Strobl LJ, Muller W, et al. Constitutive CD40 signaling in B cells selectively activates the noncanonical NF-kappaB pathway and promotes lymphomagenesis. *J Exp Med* (2008) 205(6):1317-29. doi: 10.1084/jem.20080238
155. Imtiyaz HZ, Rosenberg S, Zhang Y, Rahman ZS, Hou YJ, Manser T, et al. The Fas-associated death domain protein is required in apoptosis and TLR-induced proliferative responses in B cells. *J Immunol* (2006) 176(11):6852-61. doi: 10.4049/jimmunol.176.11.6852
156. Jacome A, Gutierrez-Martinez P, Schiavoni F, Tenaglia E, Martinez P, Rodriguez-Acebes S, et al. NSMCE2 suppresses cancer and aging in mice independently of its SUMO ligase activity. *EMBO J* (2015) 34(21):2604-19. doi: 10.15252/embj.201591829
157. Jeevan-Raj BP, Robert I, Heyer V, Page A, Wang JH, Cammas F, et al. Epigenetic tethering of AID to the donor switch region during immunoglobulin class switch recombination. *J Exp Med* (2011) 208(8):1649-60. doi: 10.1084/jem.20110118
158. Jellusova J, Cato MH, Apgar JR, Ramezani-Rad P, Leung CR, Chen C, et al. Gsk3 is a metabolic checkpoint regulator in B cells. *Nat Immunol* (2017) 18(3):303-12. doi: 10.1038/ni.3664
159. Jin HY, Gonzalez-Martin A, Miletic AV, Lai M, Knight S, Sabouri-Ghomi M, et al. Transfection of microRNA Mimics Should Be Used with Caution. *Front Genet* (2015) 6:340. doi: 10.3389/fgene.2015.00340
160. Johnson JL, Rosenthal RL, Knox JJ, Myles A, Naradikian MS, Madej J, et al. The Transcription Factor T-bet Resolves Memory B Cell Subsets with Distinct Tissue Distributions and Antibody Specificities in Mice and Humans. *Immunity* (2020) 52(5):842-55 e6. doi: 10.1016/j.immuni.2020.03.020
161. Kang JA, Kim WS, Park SG. Notch1 is an important mediator for enhancing of B-cell activation and antibody secretion by Notch ligand. *Immunology* (2014) 143(4):550-9. doi: 10.1111/imm.12333
162. Kasama Y, Mizukami T, Kusunoki H, Peveling-Oberhag J, Nishito Y, Ozawa M, et al. B-cell-intrinsic hepatitis C virus expression leads to B-cell-lymphomagenesis and induction of NF-kappaB signalling. *PLoS One* (2014) 9(3):e91373. doi: 10.1371/journal.pone.0091373
163. Kasama Y, Sekiguchi S, Saito M, Tanaka K, Satoh M, Kuwahara K, et al. Persistent expression of the full genome of hepatitis C virus in B cells induces spontaneous development of B-cell lymphomas in vivo. *Blood* (2010) 116(23):4926-33. doi: 10.1182/blood-2010-05-283358
164. Kaul S, Mittal SK, Feigenbaum L, Kruhlak MJ, Roche PA. Expression of the SNARE protein SNAP-23 is essential for cell survival. *PLoS One* (2015) 10(2):e0118311. doi: 10.1371/journal.pone.0118311
165. Kern D, Regl G, Hofbauer SW, Altenhofer P, Achatz G, Dlugosz A, et al. Hedgehog/GLI and PI3K signaling in the initiation and maintenance of chronic lymphocytic leukemia. *Oncogene* (2015) 34(42):5341-51. doi: 10.1038/onc.2014.450
166. Khiem D, Cyster JG, Schwarz JJ, Black BL. A p38 MAPK-MEF2C pathway regulates B-cell proliferation. *Proc Natl Acad Sci U S A* (2008) 105(44):17067-72. doi: 10.1073/pnas.0804868105
167. Klaewsongkram J, Yang Y, Golech S, Katz J, Kaestner KH, Weng NP. Kruppel-like factor 4 regulates B cell number and activation-induced B cell proliferation. *J Immunol* (2007) 179(7):4679-84. doi: 10.4049/jimmunol.179.7.4679
168. Knies N, Alankus B, Weilemann A, Tzankov A, Brunner K, Ruff T, et al. Lymphomagenic CARD11/BCL10/MALT1 signaling drives malignant B-cell proliferation via cooperative NF-kappaB and JNK activation. *Proc Natl Acad Sci U S A* (2015) 112(52):E7230-8. doi: 10.1073/pnas.1507459112
169. Knittel G, Liedgens P, Korovkina D, Seeger JM, Al-Baldawi Y, Al-Maarri M, et al. B-cell-specific conditional expression of Myd88p.L252P leads to the development of diffuse large B-cell lymphoma in mice. *Blood* (2016) 127(22):2732-41. doi: 10.1182/blood-2015-11-684183
170. Kobayashi M, Lin Y, Mishra A, Shelly C, Gao R, Reeh CW, et al. Bmi1 Maintains the Self-Renewal Property of Innate-like B Lymphocytes. *J Immunol* (2020) 204(12):3262-72. doi: 10.4049/jimmunol.2000030
171. Kobayashi T, Kim TS, Jacob A, Walsh MC, Kadono Y, Fuentes-Panana E, et al. TRAF6 is required for generation of the B-1a B cell compartment as well as T cell-dependent and -independent humoral immune responses. *PLoS One* (2009) 4(3):e4736. doi: 10.1371/journal.pone.0004736

172. Kober-Hasslacher M, Oh-Strauss H, Kumar D, Soberon V, Diehl C, Lech M, et al. c-Rel gain in B cells drives germinal center reactions and autoantibody production. *J Clin Invest* (2020) 130(6):3270-86. doi: 10.1172/JCI124382
173. Kracker S, Bergmann Y, Demuth I, Frappart PO, Hildebrand G, Christine R, et al. Nibrin functions in Ig class-switch recombination. *Proc Natl Acad Sci U S A* (2005) 102(5):1584-9. doi: 10.1073/pnas.0409191102
174. Kuwahara K, Fujimura S, Takahashi Y, Nakagata N, Takemori T, Aizawa S, et al. Germinal center-associated nuclear protein contributes to affinity maturation of B cell antigen receptor in T cell-dependent responses. *Proc Natl Acad Sci U S A* (2004) 101(4):1010-5. doi: 10.1073/pnas.0307609100
175. Lai M, Gonzalez-Martin A, Cooper AB, Oda H, Jin HY, Shepherd J, et al. Regulation of B-cell development and tolerance by different members of the miR-17 approximately 92 family microRNAs. *Nat Commun* (2016) 7:12207. doi: 10.1038/ncomms12207
176. Le Bouteiller M, Souilhol C, Beck-Cormier S, Stedman A, Burlen-Defranoux O, Vandormael-Pournin S, et al. Notchless-dependent ribosome synthesis is required for the maintenance of adult hematopoietic stem cells. *J Exp Med* (2013) 210(11):2351-69. doi: 10.1084/jem.20122019
177. Lehmann-Horn K, Sagan SA, Bernard CC, Sobel RA, Zamvil SS. B-cell very late antigen-4 deficiency reduces leukocyte recruitment and susceptibility to central nervous system autoimmunity. *Ann Neurol* (2015) 77(5):902-8. doi: 10.1002/ana.24387
178. Li M, Lazorchak AS, Ouyang X, Zhang H, Liu H, Arojo OA, et al. Sin1/mTORC2 regulate B cell growth and metabolism by activating mTORC1 and Myc. *Cell Mol Immunol* (2019) 16(9):757-69. doi: 10.1038/s41423-018-0185-x
179. Litzler LC, Zahn A, Meli AP, Hebert S, Patenaude AM, Methot SP, et al. PRMT5 is essential for B cell development and germinal center dynamics. *Nat Commun* (2019) 10(1):22. doi: 10.1038/s41467-018-07884-6
180. Liu B, Li Z. Endoplasmic reticulum HSP90b1 (gp96, grp94) optimizes B-cell function via chaperoning integrin and TLR but not immunoglobulin. *Blood* (2008) 112(4):1223-30. doi: 10.1182/blood-2008-03-143107
181. Liu Y, Johnson SM, Fedoriw Y, Rogers AB, Yuan H, Krishnamurthy J, et al. Expression of p16(INK4a) prevents cancer and promotes aging in lymphocytes. *Blood* (2011) 117(12):3257-67. doi: 10.1182/blood-2010-09-304402
182. Mandelbaum J, Bhagat G, Tang H, Mo T, Brahmachary M, Shen Q, et al. BLIMP1 is a tumor suppressor gene frequently disrupted in activated B cell-like diffuse large B cell lymphoma. *Cancer Cell* (2010) 18(6):568-79. doi: 10.1016/j.ccr.2010.10.030
183. Marcu-Malina V, Goldberg S, Vax E, Amariglio N, Goldstein I, Rechavi G. ADAR1 is vital for B cell lineage development in the mouse bone marrow. *Oncotarget* (2016) 7(34):54370-9. doi: 10.18632/oncotarget.11029
184. Marina-Zarate E, Perez-Garcia A, Ramiro AR. CCCTC-Binding Factor Locks Premature IgH Germline Transcription and Restrains Class Switch Recombination. *Front Immunol* (2017) 8:1076. doi: 10.3389/fimmu.2017.01076
185. McBride KM, Kil H, Mu Y, Plummer JB, Lee J, Zelazowski MJ, et al. Wwox Deletion in Mouse B Cells Leads to Genomic Instability, Neoplastic Transformation, and Monoclonal Gammopathies. *Front Oncol* (2019) 9:517. doi: 10.3389/fonc.2019.00517
186. Milanovic M, Heise N, De Silva NS, Anderson MM, Silva K, Carette A, et al. Differential requirements for the canonical NF-kappaB transcription factors c-REL and RELA during the generation and activation of mature B cells. *Immunol Cell Biol* (2017) 95(3):261-71. doi: 10.1038/icb.2016.95
187. Milasta S, Dillon CP, Sturm OE, Verbist KC, Brewer TL, Quarato G, et al. Apoptosis-Inducing-Factor-Dependent Mitochondrial Function Is Required for T Cell but Not B Cell Function. *Immunity* (2016) 44(1):88-102. doi: 10.1016/j.immuni.2015.12.002
188. Miletic AV, Anzelon-Mills AN, Mills DM, Omori SA, Pedersen IM, Shin DM, et al. Coordinate suppression of B cell lymphoma by PTEN and SHIP phosphatases. *J Exp Med* (2010) 207(11):2407-20. doi: 10.1084/jem.20091962
189. Mirlekar B, Michaud D, Lee SJ, Kren NP, Harris C, Greene K, et al. B cell-Derived IL35 Drives STAT3-Dependent CD8(+) T-cell Exclusion in Pancreatic Cancer. *Cancer Immunol Res* (2020) 8(3):292-308. doi: 10.1158/2326-6066.CIR-19-0349
190. Murga M, Lecona E, Kamileri I, Diaz M, Lugli N, Sotiriou SK, et al. POLD3 Is Haploinsufficient for DNA Replication in Mice. *Mol Cell* (2016) 63(5):877-83. doi: 10.1016/j.molcel.2016.07.007
191. Muto T, Okazaki IM, Yamada S, Tanaka Y, Kinoshita K, Muramatsu M, et al. Negative regulation of activation-induced

cytidine deaminase in B cells. *Proc Natl Acad Sci U S A* (2006) 103(8):2752-7. doi: 10.1073/pnas.0510970103
192. Onal M, Xiong J, Chen X, Thostenson JD, Almeida M, Manolagas SC, et al. Receptor activator of nuclear factor kappaB ligand (RANKL) protein expression by B lymphocytes contributes to ovariectomy-induced bone loss. *J Biol Chem* (2012) 287(35):29851-60. doi: 10.1074/jbc.M112.377945
193. Ottens K, Schneider J, Kane LP, Satterthwaite AB. PIK3IP1 Promotes Extrafollicular Class Switching in T-Dependent Immune Responses. *J Immunol* (2020) 205(8):2100-8. doi: 10.4049/jimmunol.2000584
194. Ouk C, Roland L, Gachard N, Poulain S, Oblet C, Rizzo D, et al. Continuous MYD88 Activation Is Associated With Expansion and Then Transformation of IgM Differentiating Plasma Cells. *Front Immunol* (2021) 12:641692. doi: 10.3389/fimmu.2021.641692
195. Park SG, Long M, Kang JA, Kim WS, Lee CR, Im SH, et al. The kinase PDK1 is essential for B-cell receptor mediated survival signaling. *PLoS One* (2013) 8(2):e55378. doi: 10.1371/journal.pone.0055378
196. Park SY, Wolfram P, Canty K, Harley B, Nombela-Arrieta C, Pivarnik G, et al. Focal adhesion kinase regulates the localization and retention of pro-B cells in bone marrow microenvironments. *J Immunol* (2013) 190(3):1094-102. doi: 10.4049/jimmunol.1202639
197. Pechloff K, Holch J, Ferch U, Schweneker M, Brunner K, Kremer M, et al. The fusion kinase ITK-SYK mimics a T cell receptor signal and drives oncogenesis in conditional mouse models of peripheral T cell lymphoma. *J Exp Med* (2010) 207(5):1031-44. doi: 10.1084/jem.20092042
198. Pereira JP, An J, Xu Y, Huang Y, Cyster JG. Cannabinoid receptor 2 mediates the retention of immature B cells in bone marrow sinusoids. *Nat Immunol* (2009) 10(4):403-11. doi: 10.1038/ni.1710
199. Pfeifer M, Brem R, Lippert TP, Boulianne B, Ho HN, Robinson ME, et al. SSB1/SSB2 Proteins Safeguard B Cell Development by Protecting the Genomes of B Cell Precursors. *J Immunol* (2019) 202(12):3423-33. doi: 10.4049/jimmunol.1801618
200. Polato F, Callen E, Wong N, Faryabi R, Bunting S, Chen HT, et al. CtIP-mediated resection is essential for viability and can operate independently of BRCA1. *J Exp Med* (2014) 211(6):1027-36. doi: 10.1084/jem.20131939
201. Pracht K, Meinzinger J, Schulz SR, Daum P, Corte-Real J, Hauke M, et al. miR-148a controls metabolic programming and survival of mature CD19-negative plasma cells in mice. *Eur J Immunol* (2021) 51(5):1089-109. doi: 10.1002/eji.202048993
202. Ramon S, Bancos S, Thatcher TH, Murant TI, Moshkani S, Sahler JM, et al. Peroxisome proliferator-activated receptor gamma B cell-specific-deficient mice have an impaired antibody response. *J Immunol* (2012) 189(10):4740-7. doi: 10.4049/jimmunol.1200956
203. Rivas MA, Meydan C, Chin CR, Challman MF, Kim D, Bhinder B, et al. Smc3 dosage regulates B cell transit through germinal centers and restricts their malignant transformation. *Nat Immunol* (2021) 22(2):240-53. doi: 10.1038/s41590-020-00827-8
204. Robbiani DF, Deroubaix S, Feldhahn N, Oliveira TY, Callen E, Wang Q, et al. Plasmodium Infection Promotes Genomic Instability and AID-Dependent B Cell Lymphoma. *Cell* (2015) 162(4):727-37. doi: 10.1016/j.cell.2015.07.019
205. Salinas E, Gupta A, Sifford JM, Oldenburg DG, White DW, Forrest JC. Conditional mutagenesis in vivo reveals cell type- and infection stage-specific requirements for LANA in chronic MHV68 infection. *PLoS Pathog* (2018) 14(1):e1006865. doi: 10.1371/journal.ppat.1006865
206. Sandoval GJ, Graham DB, Gmyrek GB, Akilesh HM, Fujikawa K, Sammut B, et al. Novel mechanism of tumor suppression by polarity gene discs large 1 (DLG1) revealed in a murine model of pediatric B-ALL. *Cancer Immunol Res* (2013) 1(6):426-37. doi: 10.1158/2326-6066.CIR-13-0065
207. Sanjo H, Hikida M, Aiba Y, Mori Y, Hatano N, Ogata M, et al. Extracellular signal-regulated protein kinase 2 is required for efficient generation of B cells bearing antigen-specific immunoglobulin G. *Mol Cell Biol* (2007) 27(4):1236-46. doi: 10.1128/MCB.01530-06
208. Schmidt K, Sack U, Graf R, Winkler W, Popp O, Mertins P, et al. B-Cell-Specific Myd88 L252P Expression Causes a Premalignant Gammopathy Resembling IgM MGUS. *Front Immunol* (2020) 11:602868. doi: 10.3389/fimmu.2020.602868
209. Schmidt-Supprian M, Wunderlich FT, Rajewsky K. Excision of the Frt-flanked neo (R) cassette from the CD19cre

knock-in transgene reduces Cre-mediated recombination. *Transgenic Res* (2007) 16(5):657-60. doi: 10.1007/s11248-007-9100-4
210. Schuman J, Chen Y, Podd A, Yu M, Liu HH, Wen R, et al. A critical role of TAK1 in B-cell receptor-mediated nuclear factor kappaB activation. *Blood* (2009) 113(19):4566-74. doi: 10.1182/blood-2008-08-176057
211. Sewastianik T, Jiang M, Sukhdeo K, Patel SS, Roberts K, Kang Y, et al. Constitutive Ras signaling and Ink4a/Arf inactivation cooperate during the development of B-ALL in mice. *Blood Adv* (2017) 1(25):2361-74. doi: 10.1182/bloodadvances.2017012211
212. Shi W, Bain AL, Schwer B, Al-Ejeh F, Smith C, Wong L, et al. Essential developmental, genomic stability, and tumour suppressor functions of the mouse orthologue of hSSB1/NABP2. *PLoS Genet* (2013) 9(2):e1003298. doi: 10.1371/journal.pgen.1003298
213. Shukla V, Shukla A, Joshi SS, Lu R. Interferon regulatory factor 4 attenuates Notch signaling to suppress the development of chronic lymphocytic leukemia. *Oncotarget* (2016) 7(27):41081-94. doi: 10.18632/oncotarget.9596
214. Souroullas GP, Jeck WR, Parker JS, Simon JM, Liu JY, Paulk J, et al. An oncogenic Ezh2 mutation induces tumors through global redistribution of histone 3 lysine 27 trimethylation. *Nat Med* (2016) 22(6):632-40. doi: 10.1038/nm.4092
215. Spehalski E, Capper KM, Smith CJ, Morgan MJ, Dinkelmann M, Buis J, et al. MRE11 Promotes Tumorigenesis by Facilitating Resistance to Oncogene-Induced Replication Stress. *Cancer Res* (2017) 77(19):5327-38. doi: 10.1158/0008-5472.CAN-17-1355
216. Starnes LM, Su D, Pikkupeura LM, Weinert BT, Santos MA, Mund A, et al. A PTIP-PA1 subcomplex promotes transcription for IgH class switching independently from the associated MLL3/MLL4 methyltransferase complex. *Genes Dev* (2016) 30(2):149-63. doi: 10.1101/gad.268797.115
217. Stern P, Astrof S, Erkeland SJ, Schustak J, Sharp PA, Hynes RO. A system for Cre-regulated RNA interference in vivo. *Proc Natl Acad Sci U S A* (2008) 105(37):13895-900. doi: 10.1073/pnas.0806907105
218. Suzuki A, Kaisho T, Ohishi M, Tsukio-Yamaguchi M, Tsubata T, Koni PA, et al. Critical roles of Pten in B cell homeostasis and immunoglobulin class switch recombination. *J Exp Med* (2003) 197(5):657-67. doi: 10.1084/jem.20021101
219. Tabor DE, Gould KA. Estrogen receptor alpha promotes lupus in (NZBxNZW)F1 mice in a B cell intrinsic manner. *Clin Immunol* (2017) 174:41-52. doi: 10.1016/j.clim.2016.10.011
220. Tanaka S, Ise W, Inoue T, Ito A, Ono C, Shima Y, et al. Tet2 and Tet3 in B cells are required to repress CD86 and prevent autoimmunity. *Nat Immunol* (2020) 21(8):950-61. doi: 10.1038/s41590-020-0700-y
221. Tavares RM, Turer EE, Liu CL, Advincula R, Scapini P, Rhee L, et al. The ubiquitin modifying enzyme A20 restricts B cell survival and prevents autoimmunity. *Immunity* (2010) 33(2):181-91. doi: 10.1016/j.immuni.2010.07.017
222. Thomas KR, Allenspach EJ, Camp ND, Wray-Dutra MN, Khim S, Zielinska-Kwiatkowska A, et al. Activated interleukin-7 receptor signaling drives B-cell acute lymphoblastic leukemia in mice. *Leukemia* (2021). doi: 10.1038/s41375-021-01326-x
223. Ulbricht C, Leben R, Rakhymzhan A, Kirchhoff F, Nitschke L, Radbruch H, et al. Intravital quantification reveals dynamic calcium concentration changes across B cell differentiation stages. *Elife* (2021) 10. doi: 10.7554/eLife.56020
224. Vanoaica L, Richman L, Jaworski M, Darshan D, Luther SA, Kuhn LC. Conditional deletion of ferritin h in mice reduces B and T lymphocyte populations. *PLoS One* (2014) 9(2):e89270. doi: 10.1371/journal.pone.0089270
225. Veillette A, Zhang S, Shi X, Dong Z, Davidson D, Zhong MC. SAP expression in T cells, not in B cells, is required for humoral immunity. *Proc Natl Acad Sci U S A* (2008) 105(4):1273-8. doi: 10.1073/pnas.0710698105
226. Walsh NC, Waters LR, Fowler JA, Lin M, Cunningham CR, Brooks DG, et al. LKB1 inhibition of NF-kappaB in B cells prevents T follicular helper cell differentiation and germinal center formation. *EMBO Rep* (2015) 16(6):753-68. doi: 10.15252/embr.201439505
227. Wang Y, Murakami Y, Yasui T, Wakana S, Kikutani H, Kinoshita T, et al. Significance of glycosylphosphatidylinositol-anchored protein enrichment in lipid rafts for the control of autoimmunity. *J Biol Chem* (2013) 288(35):25490-9. doi: 10.1074/jbc.M113.492611
228. Weber M, Treanor B, Depoil D, Shinohara H, Harwood NE, Hikida M, et al. Phospholipase C-gamma2 and Vav cooperate within signaling microclusters to propagate B cell spreading in response to membrane-bound antigen. *J Exp Med* (2008) 205(4):853-68. doi: 10.1084/jem.20072619

229. Westerberg LS, Dahlberg C, Baptista M, Moran CJ, Detre C, Keszei M, et al. Wiskott-Aldrich syndrome protein (WASP) and N-WASP are critical for peripheral B-cell development and function. *Blood* (2012) 119(17):3966-74. doi: 10.1182/blood-2010-09-308197
230. Wilhelmson AS, Lantero Rodriguez M, Stubelius A, Fogelstrand P, Johansson I, Buechler MB, et al. Testosterone is an endogenous regulator of BAFF and splenic B cell number. *Nat Commun* (2018) 9(1):2067. doi: 10.1038/s41467-018-04408-0
231. Wirtz T, Weber T, Kracker S, Sommermann T, Rajewsky K, Yasuda T. Mouse model for acute Epstein-Barr virus infection. *Proc Natl Acad Sci U S A* (2016) 113(48):13821-6. doi: 10.1073/pnas.1616574113
232. Xiao S, Bod L, Pochet N, Kota SB, Hu D, Madi A, et al. Checkpoint Receptor TIGIT Expressed on Tim-1(+) B Cells Regulates Tissue Inflammation. *Cell Rep* (2020) 32(2):107892. doi: 10.1016/j.celrep.2020.107892
233. Xu W, Fukuyama T, Ney PA, Wang D, Rehg J, Boyd K, et al. Global transcriptional coactivators CREB-binding protein and p300 are highly essential collectively but not individually in peripheral B cells. *Blood* (2006) 107(11):4407-16. doi: 10.1182/blood-2005-08-3263
234. Yagi T, Giallourakis C, Mohanty S, Scheidig C, Shen J, Zheng H, et al. Defective signal transduction in B lymphocytes lacking presenilin proteins. *Proc Natl Acad Sci U S A* (2008) 105(3):979-84. doi: 10.1073/pnas.0707755105
235. Yang D, Sun Y, Chen J, Zhang Y, Fan S, Huang M, et al. REV7 is required for processing AID initiated DNA lesions in activated B cells. *Nat Commun* (2020) 11(1):2812. doi: 10.1038/s41467-020-16632-8
236. Yang L, Yildirim E, Kirby JE, Press W, Lee JT. Widespread organ tolerance to Xist loss and X reactivation except under chronic stress in the gut. *Proc Natl Acad Sci U S A* (2020) 117(8):4262-72. doi: 10.1073/pnas.1917203117
237. Yang Y, Li X, Ma Z, Wang C, Yang Q, Byrne-Steele M, et al. CTLA-4 expression by B-1a B cells is essential for immune tolerance. *Nat Commun* (2021) 12(1):525. doi: 10.1038/s41467-020-20874-x
238. Ye M, Ermakova O, Graf T. PU.1 is not strictly required for B cell development and its absence induces a B-2 to B-1 cell switch. *J Exp Med* (2005) 202(10):1411-22. doi: 10.1084/jem.20051089
239. Yen WF, Sharma R, Cols M, Lau CM, Chaudhry A, Chowdhury P, et al. Distinct Requirements of CHD4 during B Cell Development and Antibody Response. *Cell Rep* (2019) 27(5):1472-86 e5. doi: 10.1016/j.celrep.2019.04.011
240. Yu Q, Quinn WJ, 3rd, Salay T, Crowley JE, Cancro MP, Sen JM. Role of beta-catenin in B cell development and function. *J Immunol* (2008) 181(6):3777-83. doi: 10.4049/jimmunol.181.6.3777
241. Zhang J, Dominguez-Sola D, Hussein S, Lee JE, Holmes AB, Bansal M, et al. Disruption of KMT2D perturbs germinal center B cell development and promotes lymphomagenesis. *Nat Med* (2015) 21(10):1190-8. doi: 10.1038/nm.3940
242. Zhang J, Vlasevska S, Wells VA, Nataraj S, Holmes AB, Duval R, et al. The CREBBP Acetyltransferase Is a Haploinsufficient Tumor Suppressor in B-cell Lymphoma. *Cancer Discov* (2017) 7(3):322-37. doi: 10.1158/2159-8290.CD-16-1417
243. Zhang S, Pruitt M, Tran D, Du Bois W, Zhang K, Patel R, et al. B cell-specific deficiencies in mTOR limit humoral immune responses. *J Immunol* (2013) 191(4):1692-703. doi: 10.4049/jimmunol.1201767
244. Zhang S, Zhou X, Lang RA, Guo F. RhoA of the Rho family small GTPases is essential for B lymphocyte development. *PLoS One* (2012) 7(3):e33773. doi: 10.1371/journal.pone.0033773
245. Zhou X, Zhu X, Li C, Li Y, Ye Z, Shapiro VS, et al. Stearoyl-CoA Desaturase-Mediated Monounsaturated Fatty Acid Availability Supports Humoral Immunity. *Cell Rep* (2021) 34(1):108601. doi: 10.1016/j.celrep.2020.108601
246. Zhu Z, Shukla A, Ramezani-Rad P, Apgar JR, Rickert RC. The AKT isoforms 1 and 2 drive B cell fate decisions during the germinal center response. *Life Sci Alliance* (2019) 2(6). doi: 10.26508/lsa.201900506

III. Studies with undefined control mice

247. Barrow F, Khan S, Fredrickson G, Wang H, Dietsche K, Parthiban P, et al. Microbiota-Driven Activation of Intrahepatic B Cells Aggravates NASH Through Innate and Adaptive Signaling. *Hepatology* (2021) 74(2):704-22. doi: 10.1002/hep.31755
248. Belyea BC, Xu F, Sequeira-Lopez MLS, Gomez RA. Leukemia development initiated by deletion of RBP-J: mouse strain, deletion efficiency and cell of origin. *Dis Model Mech* (2018) 11(12). doi: 10.1242/dmm.036731
249. Kontoyiannis D, Boulougouris G, Manoloukos M, Armaka M, Apostolaki M, Pizarro T, et al. Genetic dissection of the

cellular pathways and signaling mechanisms in modeled tumor necrosis factor-induced Crohn's-like inflammatory bowel disease. *J Exp Med* (2002) 196(12):1563-74. doi: 10.1084/jem.20020281
250. Lehmann-Horn K, Sagan SA, Winger RC, Spencer CM, Bernard CC, Sobel RA, et al. CNS accumulation of regulatory B cells is VLA-4-dependent. *Neurol Neuroimmunol Neuroinflamm* (2016) 3(2):e212. doi: 10.1212/NXI.0000000000000212
251. Park KS, Bayles I, Szlachta-McGinn A, Paul J, Boiko J, Santos P, et al. Transcription elongation factor ELL2 drives Ig secretory-specific mRNA production and the unfolded protein response. *J Immunol* (2014) 193(9):4663-74. doi: 10.4049/jimmunol.1401608
252. Pasparakis M, Schmidt-Supprian M, Rajewsky K. IkappaB kinase signaling is essential for maintenance of mature B cells. *J Exp Med* (2002) 196(6):743-52. doi: 10.1084/jem.20020907
253. Patzelt T, Keppler SJ, Gorka O, Thoene S, Wartewig T, Reth M, et al. Foxp1 controls mature B cell survival and the development of follicular and B-1 B cells. *Proc Natl Acad Sci U S A* (2018) 115(12):3120-5. doi: 10.1073/pnas.1711335115
254. Waisertreiger I, Popovich K, Block M, Anderson KR, Barlow JH. Visualizing locus-specific sister chromatid exchange reveals differential patterns of replication stress-induced fragile site breakage. *Oncogene* (2020) 39(6):1260-72. doi: 10.1038/s41388-019-1054-5
255. Wu JL, Chiang MF, Hsu PH, Tsai DY, Hung KH, Wang YH, et al. O-GlcNAcylation is required for B cell homeostasis and antibody responses. *Nat Commun* (2017) 8(1):1854. doi: 10.1038/s41467-017-01677-z
256. Zhang P, Zhao Y, Sun XH. Notch-regulated periphery B cell differentiation involves suppression of E protein function. *J Immunol* (2013) 191(2):726-36. doi: 10.4049/jimmunol.1202134
