# Supplementary material for: Altered Phenotype and Enhanced Antibody-Producing Ability of Peripheral B Cells in Mice with Cd19-Driven Cre Expression
Source: Cells. 2022 Feb 16;11(4):700. doi: 10.3390/cells11040700 (PMC8870415; doi:10.3390/cells11040700)
Supplement: Supplementary file 1 [file cells-11-00700-s001.zip › Supplementary File S2.pdf]

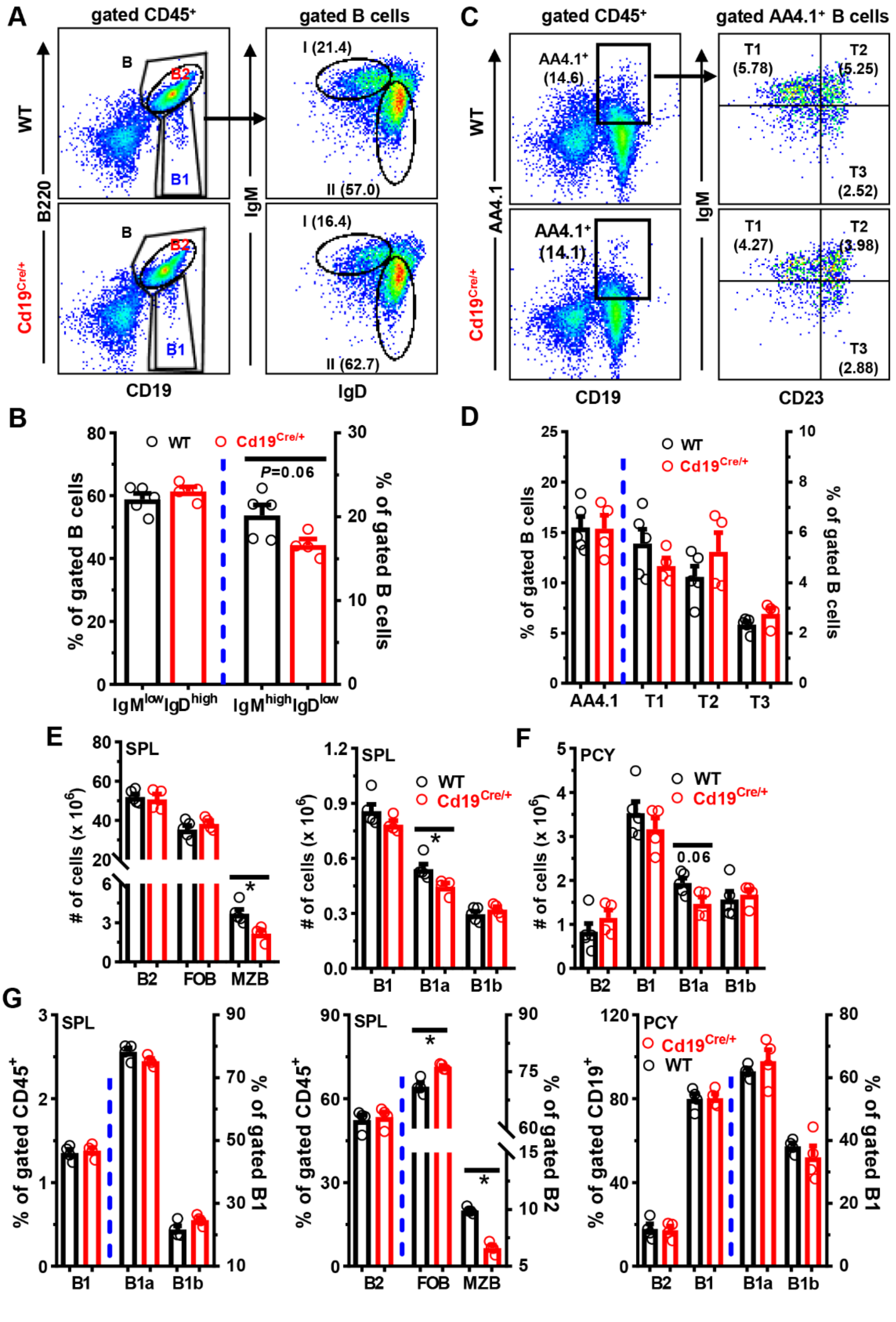

Supplementary Figure S1. Disturbed phenotypes of peripheral B cells in Cd19$^{Cre/+}$ mice. Cells from the spleens of naive mice were stained with antibodies against CD45/B220/CD19/IgD & IgM (**A&B**) or CD45/CD19/CD23/AA4.1 & IgM (**C&D**) followed by analyses on FACS. **A&C,** representative FACS plots showing the gating strategies for B (CD19^{+}), B2 (CD19^{+}B220^{high})/B1 (CD19^{+}B220^{low}) (*left panel* in **A**), or IgM^{high}IgD^{low} (I) & IgM^{low}IgD^{high} (II) cells within gated CD19^{+} B cells (*right panel* in **A**), or transitional T1 (CD19^{+}AA4.1^{+}IgM^{high}CD23^{low}), T2 (CD19^{+}AA4.1^{+}IgM^{high}CD23^{high}) & T3 (CD19^{+}AA4.1^{+}IgM^{low}CD23^{high}) B cells (**C**) in WT *vs.* Cd19$^{Cre/+}$ mice. Numbers in brackets indicated the percentages of gated subsets within B cells. **B&D**, bar graphs showing the percent of IgM^{high}IgD^{low} & IgM^{low}IgD^{high} (**B**) or AA4.1^{+}, T1, T2 & T3 (**D**) cells within gated B cells in spleens of mice. **E&F**, numbers of indicated B-cell subsets in the spleens (SPL, **E**) or peritoneal cavities (PCY, **F**) of mice (female, 8-10 weeks; n=5/4 for WT/Cd19$^{Cre/+}$ group, respectively). **G**, bar graphs showing the percent of indicated B-cell subsets in the SPL or PCY of aged WT *vs.* Cd19$^{Cre/+}$ mice (female, 16 weeks old, n=4 for each genotype). B-cell subsets were gated as illustrated in Figure 2A&C. Each symbol represented one single mouse, and results were expressed as mean ± SEM (**B, D-G**). $^{*}P < 0.05$.

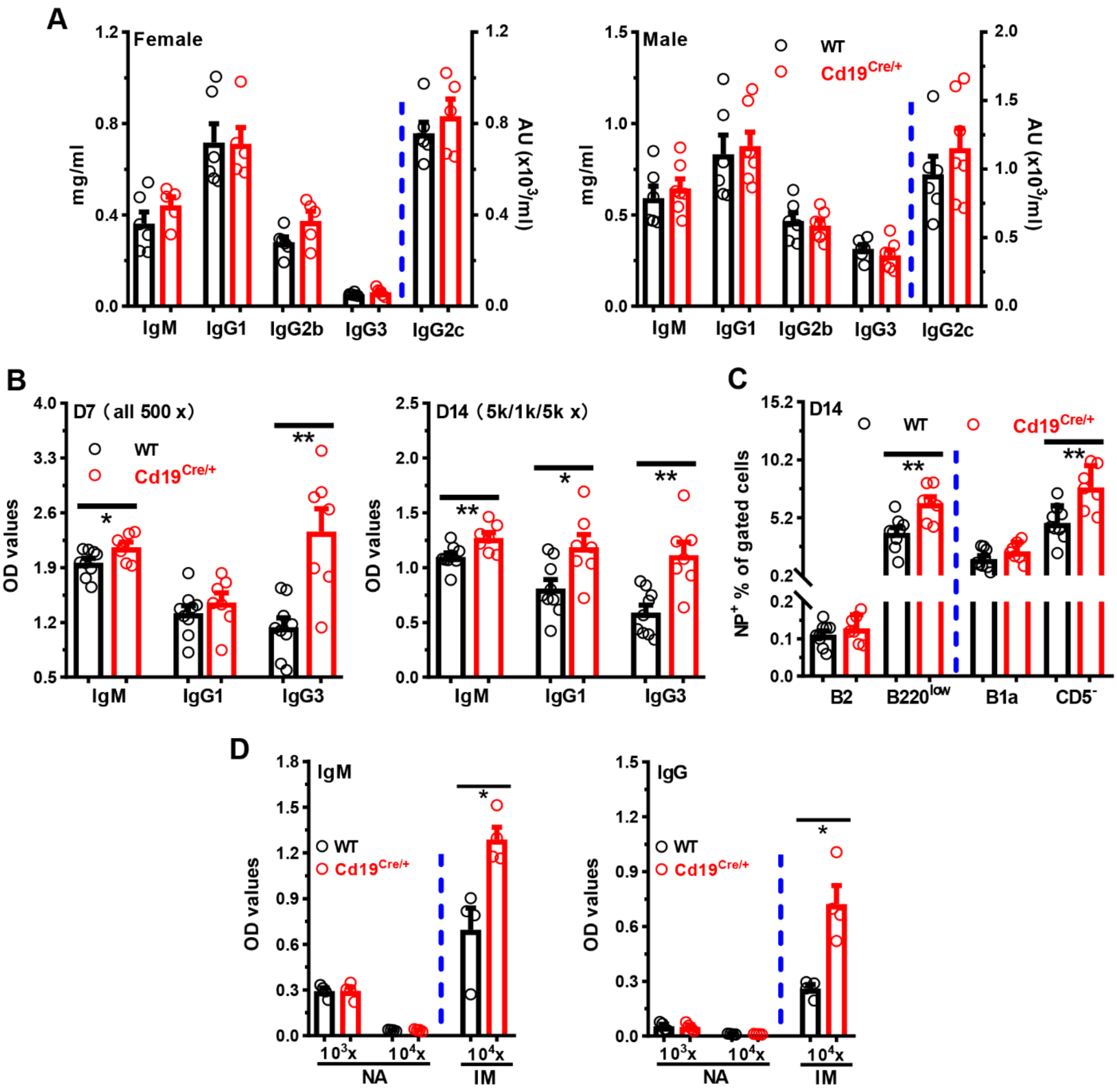

Supplementary Figure S2. Normal baseline sera antibody levels but enhanced humoral immune responses after immunization in Cd19$^{Cre/+}$ mice. A, bar graphs showing levels of total IgM, IgG1, IgG2b, IgG3 and IgG2c in sera of female (~ 8 weeks old, n=6/5 for WT/Cd19$^{Cre/+}$, respectively) or male (8-10 weeks old, n=6/7 for WT/ Cd19$^{Cre/+}$, respectively) mice. **B**, bar graphs showing levels of NP-specific IgM, IgG1 & IgG3 in sera of male WT or Cd19$^{Cre/+}$ mice on D7 & D14 post immunization. **C**, a bar graph showing the percent of NP^{+} cells within gated blood B2 (CD19^{+}B220^{high}), B220^{low} (CD19^{+}B220^{low}), B1a (CD19^{+}B220^{low}CD5^{+}) or CD5^{-}(CD19^{+}B220^{low}CD5^{-}) B cells in male mice on D14 post immunization. Mice (male, 11-13 weeks old, n=9/7 for WT/Cd19$^{Cre/+}$, respectively) were immunized with NP-Ficoll (10 μg/100μl/mouse in PBS) intraperitoneally on D0 (**B&C**). **D**, bar graphs showing levels of NP-specific IgM & IgG in sera of naive (NA) or NP-Ficoll immunized (IM) WT or Cd19$^{Cre/+}$ mice (male, ~12 weeks old, n=4 for each genotype). Numbers in the upper left corner (**B**) or below bars (**D**) indicated the dilutions of sera for detection of each antibody subtype. Each symbol represented one single mouse of the indicated genotype, and results were expressed as mean ±SEM. * $P < 0.05$; ** $P < 0.01$.

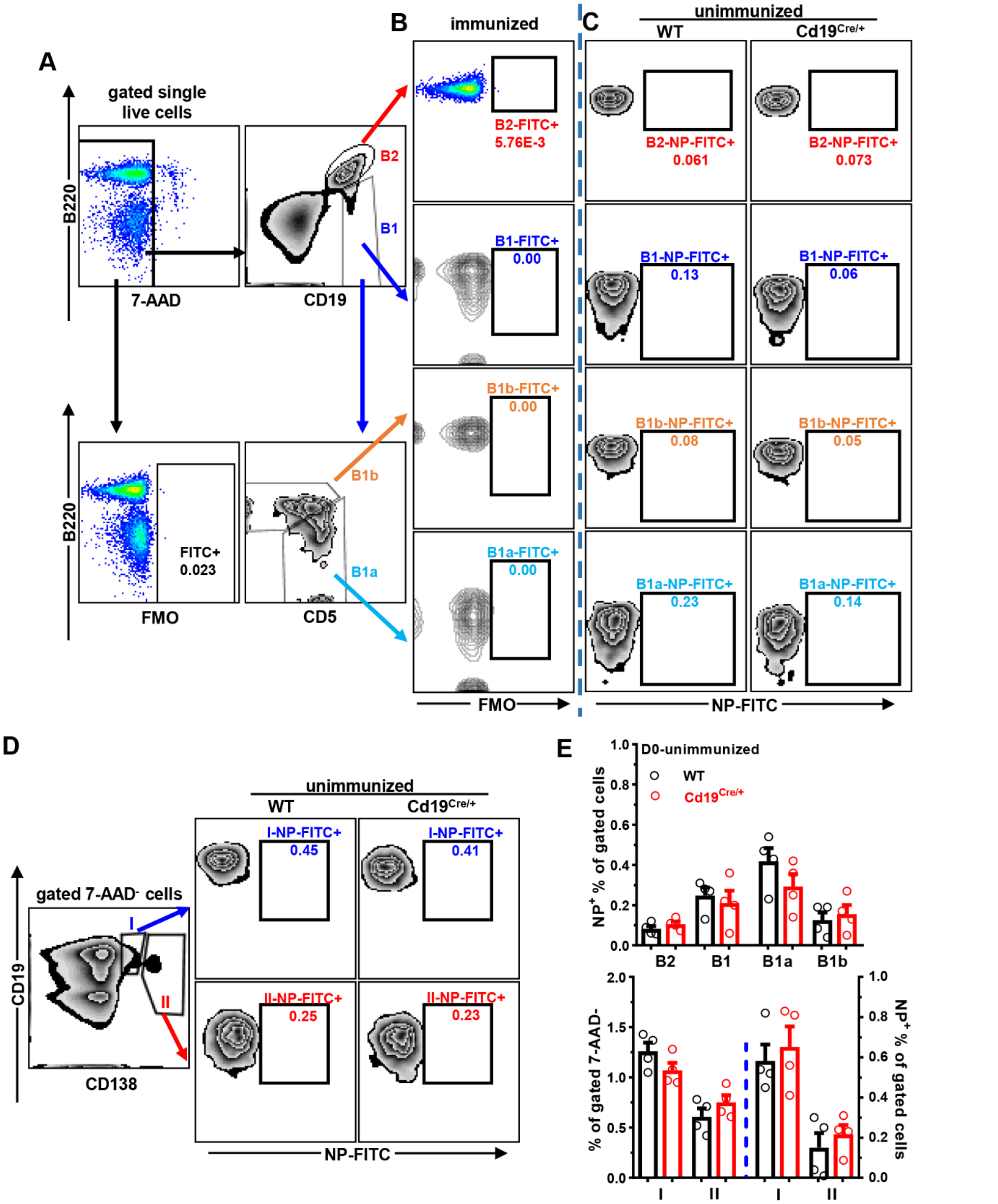

Supplementary Figure S3. Comparable background stainings of NP-Ficoll in unimmunized WT *vs.* Cd19$^{Cre/+}$ mice. Splenic or blood cells were stained with antibodies against CD45/B220/CD19/CD5 (**A-C**) or CD45/CD19/CD138 (**D**) plus 7-AAD and NP-FITC. **A**, representative FACS plots showing the gating strategies for B2, B1, B1a or B1b cells. **B**, representative FACS plots illustrating the signal in FITC channel in cells from an immunized WT mice (depicted in Figure 4) stained with all antibodies but without NP-FITC (FMO, fluorescence minus one/FITC). **C&D**, representative FACS plots showing percent of NP-FITC^{+} cells within gated B2 (B2-NP-FITC+), B1 (B1-NP-FITC+), B1a (B1a-NP-FITC+) & B1b (B1b-NP-FITC+) (**C**), or gated CD19^{+}CD138^{low} (I-NP-FITC+)/CD19^{low}CD138^{high} (II-NP-FITC+) (**D**) in unimmunized WT *vs.* Cd19$^{Cre/+}$ mice. **E**, bar graphs showing the percent of NP^{+} cells within gated B-cell subsets or frequencies of CD19^{+}CD138^{low} (I)/CD19^{low}CD138^{high} (II) in the two groups of mice (female, 12 weeks old, n=4 for each genotype). Each symbol represented one single mouse of the indicated genotype, and results were expressed as mean ±SEM (**E**).

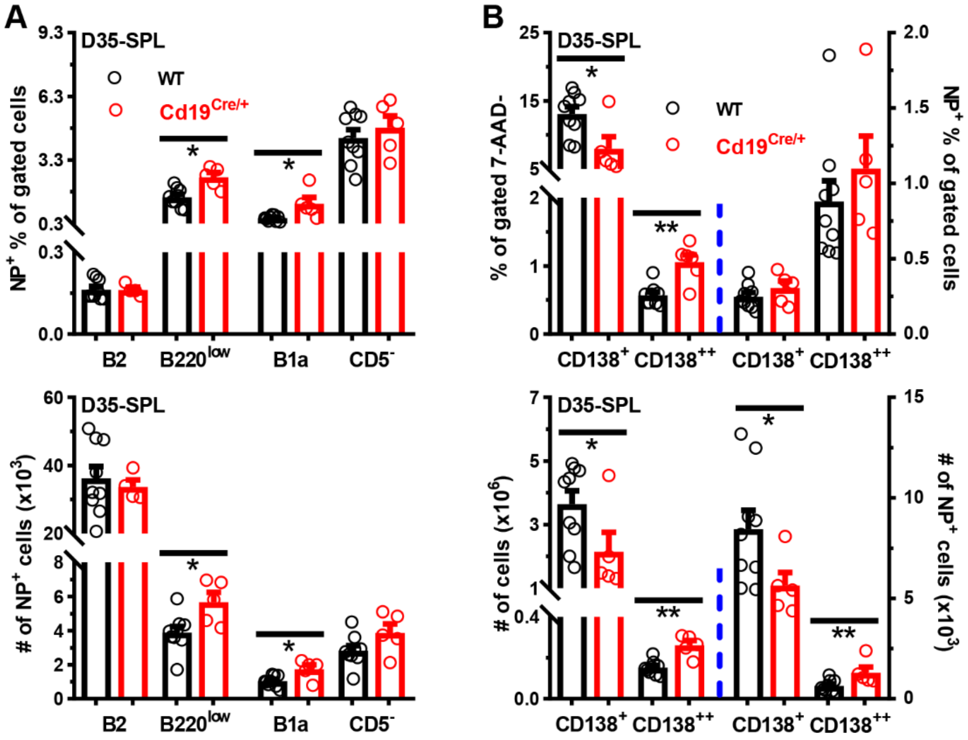

Supplementary Figure S4. Increased numbers of NP$^+$ B cells in spleens of Cd19$^{Cre/+}$ mice immunized with NP-Ficoll. Mice (female, ~ 10 weeks of age) were immunized with NP-Ficoll (10 µg/100µl/mouse in PBS) intraperitoneally on D0. Cells in spleens (SPL) on D35 post immunization were stained with 7-AAD, NP-FITC plus antibodies against B220/CD19/CD5 & CD138. **A,** bar graphs showing the percent (*upper panel*) and numbers (*low panel*) of NP$^+$ cells within gated CD19$^+$B220^{high} B2, CD19$^+$B220^{low} (B220^{low}), CD19$^+$B220^{low}CD5$^+$ B1a or CD19$^+$B220^{low}CD5$^-$ (CD5$^-$) B cells in WT *vs.* Cd19$^{Cre/+}$ mice. **B,** bar graphs showing the percent (*upper panel*) and numbers (*low panel*) of CD19$^+$CD138^{low} (CD138$^+$)/CD19^{low}CD138^{high} (CD138^{++}) or NP$^+$ cells within gated CD138$^+$/CD138^{++} B cells in the two groups of mice. Each symbol represented one single mouse of the indicated genotype (n=9/5 for WT/Cd19$^{Cre/+}$ group, respectively), and results were expressed as mean ± SEM. * $P < 0.05$; ** $P < 0.01$.

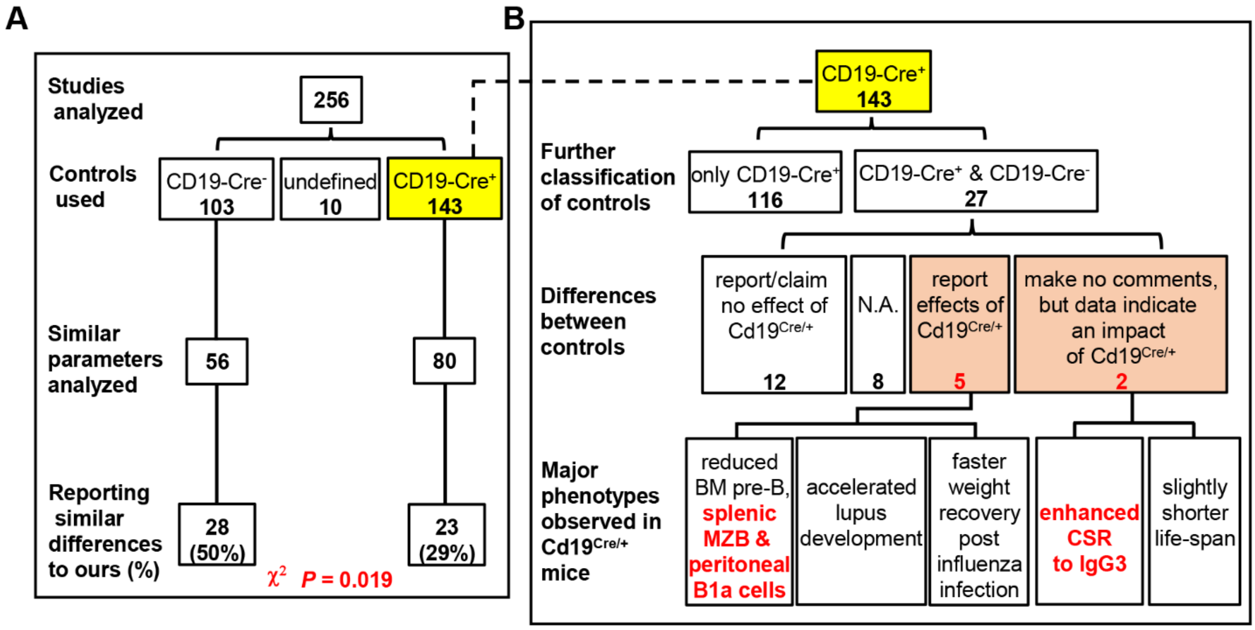

Supplementary Figure S5. Survey of studies. In total 256 articles (**Supplementary File 1**) where the *Cd19*$^{Cre/+}$ transgene was used to delete loxp-flanked sequence in B cells were analyzed, among which 10 studies contained no information on control mice or used pooled mice with different genotypes as the control group (undefined in **A**). CD19-Cre^{-} control mice, including WT (*gene*$^{+/+}$), floxed hemizygotes (*gene*$^{fl/+}$) or homozygotes (*gene*$^{fl/fl}$), were used in 103 studies, whereas mice with one *Cd19*$^{Cre/+}$ knockin allele (CD19-Cre^{+}) in combination with WT or floxed heterozygous (*gene*$^{fl/+}$) alleles were included as controls in the remaining 143 papers. Within these 246 articles, 136 (56 out of 103 using CD19-Cre^{-} and 80 out of 143 using CD19-Cre^{+} mice as controls, respectively) studies contained the necessary information allowing for comparisons between the control and *Cd19*$^{Cre/+}$*gene*$^{fl/+}$ or *Cd19*$^{Cre/+}$*gene*$^{fl/fl}$ mice in at least one of the following parameters: percent/numbers of MZB & B1a cells, or antibody levels *in vitro/vivo*. At least one of the following differences was present for the results reported to be considered similar (to the phenotype of Cd19$^{Cre/+}$ mice described in this manuscript): reduced MZB or B1a cells, and increased antibody levels in sera or culture supernatants. χ^2 tests were employed to compare the results from published articles using CD19-Cre^{-} (56) or CD19-Cre^{+} (80) mice as controls (**A**). **B**, major impacts of the single *Cd19*$^{Cre/+}$ knockin allele reported in the literatures. Among 27 studies using both CD19-Cre^{+} & CD19-Cre^{-} mice as controls, 5 studies reported significant differences between the two types of controls used, while data in two other publications indicated an augmented CSR to IgG3 of B cells or a reduced long-term survival rate in mice with a *Cd19*$^{Cre/+}$ knockin allele. N.A.: no assessment on the impact of *Cd19*$^{Cre/+}$ transgene could be made as *Cd19*$^{Cre/+}$*gene*$^{fl/+}$ heterozygotes and CD19-Cre^{-} mice were used as controls.
